# Recent progress in highly effective electrocoagulation-coupled systems for advanced wastewater treatment

**DOI:** 10.1016/j.isci.2025.111965

**Published:** 2025-02-06

**Authors:** Thi Kim Cuong Phu, Phi Long Nguyen, Thi Viet Bac Phung

**Affiliations:** 1 Center for Environmental Intelligence and College of Engineering and Computer Science, VinUniversity, Hanoi 100000, Vietnam; 2 Faculty of Electrical Engineering, Hanoi University of Industry, Hanoi 100000, Vietnam

**Keywords:** Chemical Engineering, Environmental engineering, Water geochemistry, Water resources engineering

## Abstract

Electrocoagulation (EC) has been a well-known technology for wastewater treatment over the past centuries, owing to its straightforward equipment requirements and highly effective contaminant removal efficiency. This literature review emphasizes the influence of several input variables in the EC system such as electrode materials, applied current, pH, supporting electrolyte, and inner-electrode distance on effluent removal efficiency and energy consumption. Besides that, depending on the intrinsic properties of effluents, EC is recommended to hybridize with other methods such as physical-, biological-, chemical-, and electrochemical methods in order to enhance removal performance and reduce energy consumption. Subsequently, a comprehensive analysis of EC performance is presented, including power consumption, and evaluation of the synergistic effect of multiple input variables using statistical methods. Finally, this review discusses future perspectives such as the environmentally friendly utilization of post-EC treated sludges, the development of renewable energy-driven EC systems, and the challenges of EC management by artificial intelligence.

## Graphical abstract

**Figure:**
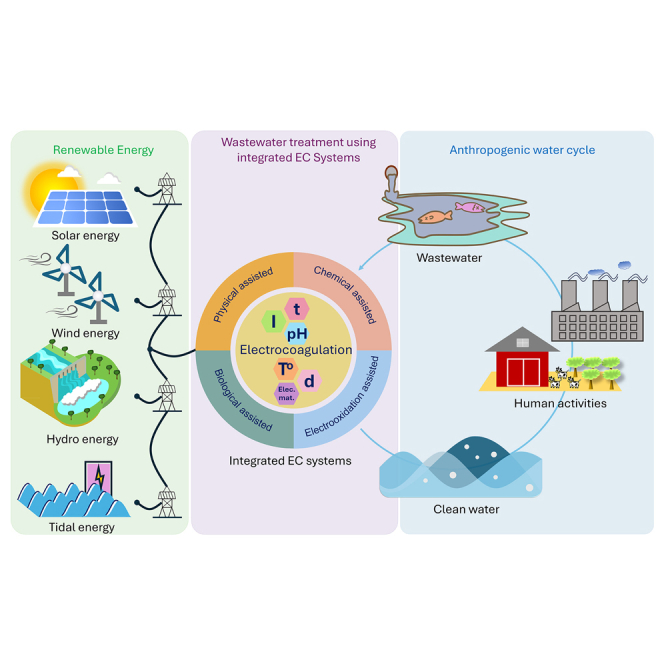

## Introduction

Water is an indispensable resource for nurturing human well-being and the diversity of Earth’s ecosystems.^1^ Various anthropogenic activities, including agriculture, livestock husbandry, industrial processes, energy production, and daily domestic tasks, heavily depend on the availability of water that meets specific quality standards.^2^ Despite Earth’s vast water resources, totaling 1.4 billion cubic kilometers, only 0.5% is fresh and readily accessible.^3^ However, human activities have significantly disrupted the natural water cycle, leading to a shortage of water in both quality and quantity.^4^ Consequently, approximately two-thirds of the global population currently faces water scarcity, and projections indicate that by 2030, around 40% of the annual water demand will surpass the available supply.^5^ Although sufficient freshwater is available globally each year to meet human needs, regional and temporal variations in water availability and demand cause water scarcity in different parts of the world at various times.^6^ Therefore, it is urgent to ensure the sustainable utilization of water and develop effective wastewater treatment methods for reusing wastewater in water-depleted regions caused by human activities, and address wastewater discharge issues.^7^

The ultimate goal of wastewater treatment methods is to completely remove contaminants from water to meet the standard requirements. Substantial research has been dedicated to enhancing the performance of various treatment methods, including physical,^8^^,^^9^^,^^10^ biological,^11^^,^^12^ chemical,^13^ and electrochemical approaches.^14^^,^^15^ Each of these methods offers distinct advantages and limitations, highlighting the need for ongoing improvements and optimization to enable their broader application in water purification. For example, chemical coagulation (CC) with its simple and cost-effective operation can effectively remove various effluents; however, solving post-treatment sludge in environmentally friendly ways is still unsatisfactory.

Currently, the development of biomaterials for adsorption methods in wastewater treatment is gaining popularity, but the treated water quality has not met the safety standards required for potable water. Therefore, effectively removing pollutants at an affordable operational cost is compulsory for the widespread adoption of wastewater treatment technologies.

**Figure 1:**
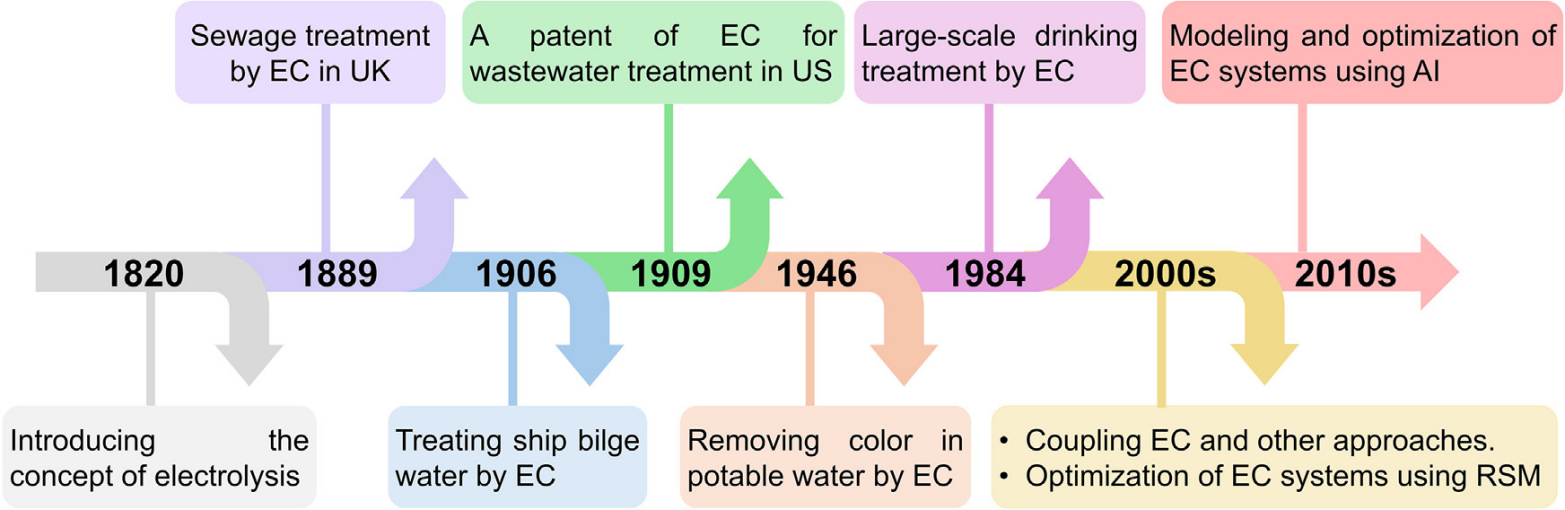
Timeline of EC research in wastewater treatment

Since the concept of electrolysis was introduced by the renowned British scientist Michael Faraday, research on electrocoagulation (EC) systems has experienced sustained growth, primarily focusing on their application in water treatment processes. However, in the first half of the 20th century, EC was less appealing as a water treatment technology compared to conventional methods due to limited electricity availability and high operational costs. With advancements in energy conversion technology, EC became more widely adopted for commercial-scale water treatment applications with affordable costs by the late 20^th^ century and into the 21^st^ century. Additionally, the optimization of EC systems has been addressed through mathematical approaches, and the integration of artificial intelligence (AI) for modeling EC systems has significantly improved the accuracy of predictions for the nonlinear relationships between input and output variables (Figure 1).^16^

**Figure 2:**
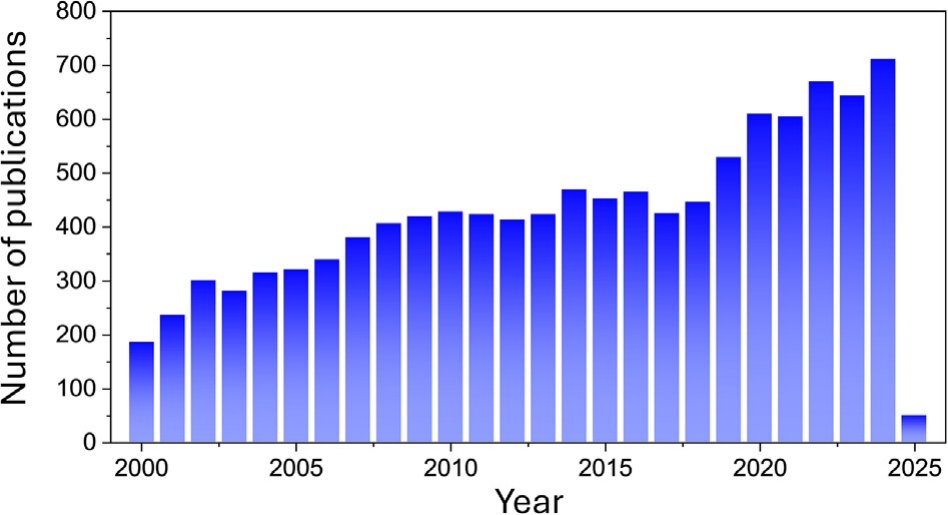
Annual publications of electrocoagulation in various fields The literature search was conducted using Elsevier’s Scopus database with the keyword “electrocoagulation”.

Since the 2000s, publications on EC have gradually increased, with its primary application being wastewater treatment (Figure 2). EC has gained significant attention as a promising technique for treating wastewater with a wide range of contaminants, owing to its ease of operation, low-cost infrastructure, and high removal efficiency. From both economic and technical perspectives, while developed countries have made substantial advancements in wastewater treatment for industrial and domestic purposes, developing and underdeveloped nations continue to face persistent challenges, such as limited access to advanced technologies and high costs. Therefore, the development and integration of EC with other treatment approaches are crucial to facilitating its widespread implementation in wastewater treatment solutions globally. This review highlights the influence of input variables on output results in EC systems, the effectiveness of EC in removing various contaminants from different wastewater sources, and the benefits of coupling EC with other approaches, including physical, chemical, biological, and electrochemical methods. Finally, this review discusses the advancements and challenges in modeling and predicting EC systems using AI.

### Background

**Figure 3:**
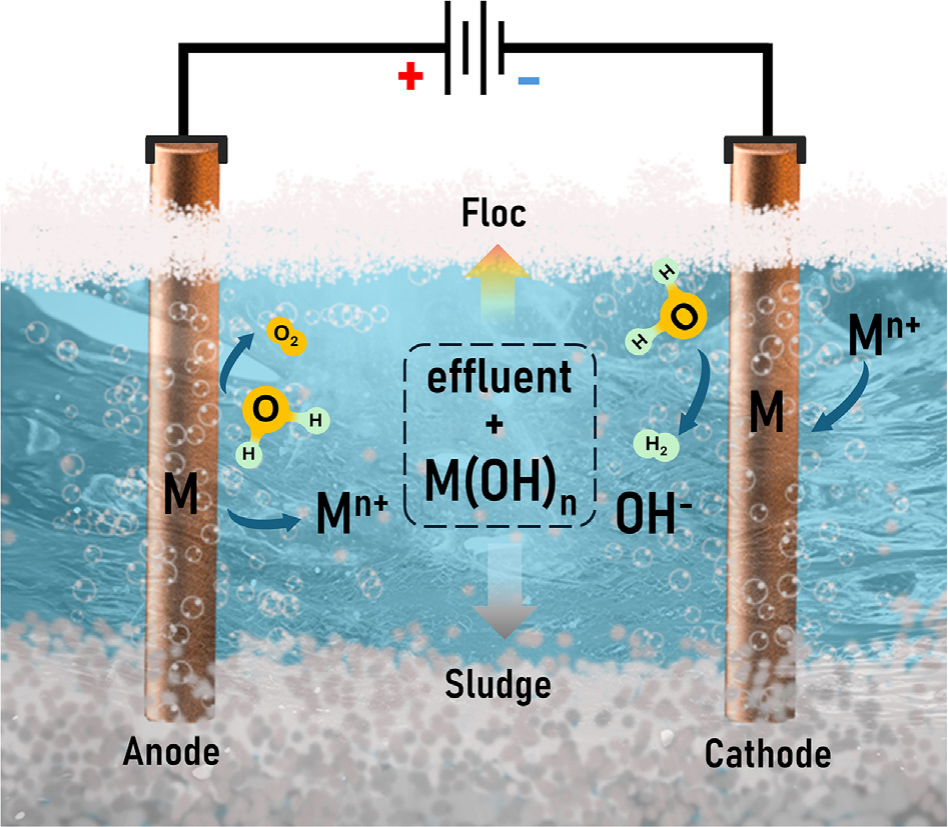
Schematic illustration of the electrocoagulation cell

As illustrated in Figure 3, EC involves processes that dissolve the metal anode (M) via oxidation through the application of an external power source potential, resulting in the *in-situ* production of coagulants, as described in Equation 1.^17^ Simultaneously, oxygen and hydrogen gases are generated at the anode and cathode, respectively (Equations 2 and 3), which aid in the flotation of contaminants. Metal ions released from the dissolving anode material react with hydroxyl ions produced at the cathode, forming hydroxides that serve as effective adsorbents. Iron and aluminum are the most commonly used anode materials due to their ability to form polyvalent ions and various hydrolysis products, which depend on the solution’s pH and the potential formation of polynuclear complexes. These coagulants interact with other contaminants in the wastewater, producing flocs (Equation 4) that can be efficiently separated by sedimentation or flotation.^18^ When the concentration of ions released from the anode becomes sufficient to induce the flocculation of all suspended particles, the remaining concentration of contaminants in the effluent is minimized.^19^

*Anode*

(Equation 1) $$M\;\to \;M^{n+}+ne^{-}$$

(Equation 2) $$2H_{2}O\;\to \;4H^{+}+O_{2}+4e^{-}$$

*Cathode*

(Equation 3) $$2H_{2}O+2e^{-}\;\to \;H_{2}+2{\text{OH}}^{-}$$

*The overall reaction*

(Equation 4) $$M^{n+}+{n\text{OH}}^{-}\;\to \;M{\left(\text{OH}\right)}_{n}$$

This method integrates the benefits of coagulation, flotation, and electrochemical processes.^20^ Compared to CC, EC exhibits reduced coagulant usage and enhanced efficacy in waste elimination.^21^ The main difference between EC and CC is the ongoing *in situ* formation of adsorbent particles in EC, which promotes the sweep flocculation effect.^22^ Although sweep flocculation is proficient in eliminating colloidal particles, it is less effective for heavy metal ions.^23^^,^^24^ Consequently, for metallic ions and dissolved organic compounds, sweep flocculation likely involves an adsorption process. These substances must initially adhere to the floc surfaces before becoming incorporated into the expanding porous matrix of the flocs.^25^ Therefore, adsorption is considered the predominant mechanism for the removal of heavy metals and ionic organic components. EC technology has also been proposed as an effective decentralized water treatment solution, with the potential for easy deployment in renewable-driven EC portable units for use in energy-depleted areas.^26^^,^^27^ Although the EC process offers several benefits, it also has certain limitations that may lead to less-than-optimal wastewater treatment outcomes (Table 1). To address these drawbacks, coupling EC with other approaches offers a particularly compelling strategy to ensure effluent concentrations comply with regulatory water safety standards.^28^^,^^29^^,^^30^ Furthermore, using EC for wastewater treatment powered by renewable electricity could simultaneously reduce carbon footprints and address environmental issues.^31^^,^^32^ This review will provide a comprehensive overview of the advancements in EC-based wastewater treatment technologies, and examine the challenges faced in their application.

**Table 1:** Advantages and disadvantages of the EC process

Advantages	Disadvantages
Efficient elimination of a broad range of contaminants.^33^ Limited use of chemical reagents, straightforward equipment, simple automation control, rapid sedimentation of flocs, minimal sludge generation, and low levels of secondary pollutants.	Reduced EC performance in treating contaminants with low conductivity.^33^ Electrode passivation, hazardous sludge production, frequent replacement of sacrificial anodes, and high electricity costs in regions with limited access to electricity.^34^

### Several parameters of the EC system

The effectiveness of EC in pollutant removal is influenced by multiple input variables, including electrode materials, current density, pH level, interelectrode spacing, electrolysis duration, the presence of supporting electrolytes, and type of electricity sources.

#### Electrode materials

**Figure 4:**
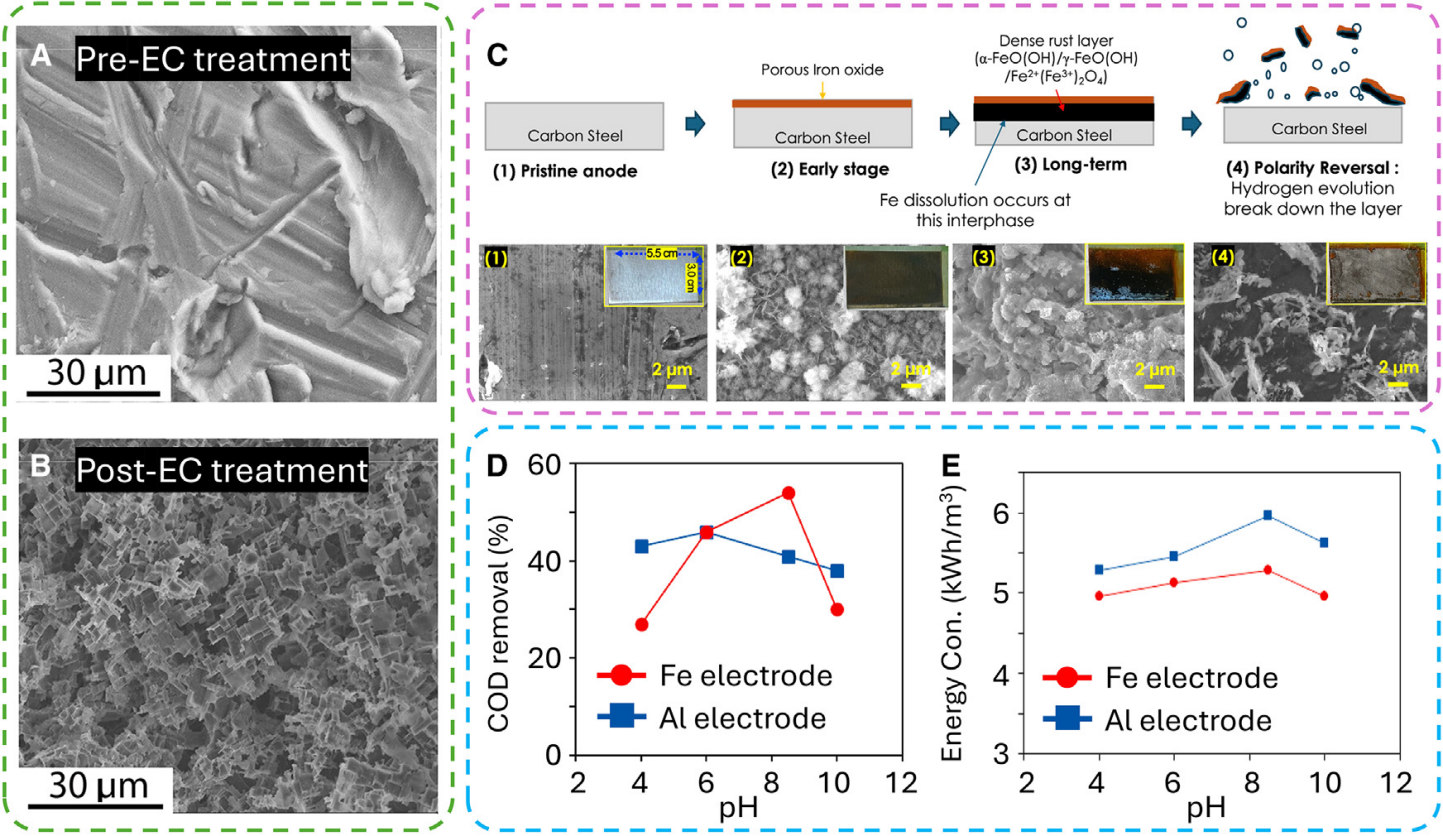
The effect of electrode materials to EC performance (A and B) SEM images of (A) pre-treated and (B) post-treated Al electrode of the anode Al surface (passivation film) when operating at pH 11 in electrolyte with different dyes.^34^ (C) Proposed mechanism of fouling layer formation on the anode during EC reaction and polarity reversal for surface cleaning: (1) pristine anode, (2) porous iron oxide layer formation at the early stage, (3) thick and dense black rust layer (containing goethite, lepidocrocite, and magnetite with passivating effects) formation during long-term performance, (4) film-free area after polarity reversal.^39^ Insets are photos of Fe electrode (i.e., carbon steel, 3.0 cm × 5.5 cm) after extended EC operation. The removal efficiency of (D) COD and (E) energy consumptions versus pH for the electrodes (5 mA/cm^2^ and 90 min).^40^

Aluminum and iron materials are predominantly employed as EC sacrificial electrodes due to their effectiveness, availability, low toxicity, and relatively affordable cost.^15^^,^^35^ Aluminum is most highly suitable for use as electrodes in the EC process due to its ability to undergo electro-dissolution and its excellent coagulation properties, which result from the formation of aluminum hydroxide flocs that effectively adsorb and neutralize contaminants.^36^ Subsequently, due to corrosion reactions at the anode, etched and highly porous morphologies were observed on the Al surface (Figures 4A and 4B).^34^^,^^37^ Besides that, the use of aluminum scrap as electrodes in EC is being investigated to improve the material reuse and sustainability of this approach.^38^ As a result, employing scrap metals as sacrificial electrodes not only lowers operational expenses but also offers a recycling avenue for metallic waste. For example, Abir et al. demonstrated an effective and economical EC system utilizing aluminum scrap electrodes, which achieved a methylene blue removal efficiency of 97.81% while maintaining relatively low energy consumption.^38^ This method paves the way for reusing waste materials in wastewater treatment, contributing to sustainable environmental conservation.

Iron is another commonly employed electrode material in EC systems with several research has employed iron as a sacrificial electrode in EC systems to remove various contaminants in both industry and domestic wastewater. When an electrical potential is applied, the sacrificial anode dissolves, releasing iron cations (Fe^2+^). These ferrous ions are subsequently oxidized by dissolved oxygen in the solution to produce ferric ions (Fe^3+^). For example, Jang et al. provided a comprehensive analysis of the surface fouling process associated with iron electrodes (Figure 4C).^39^ Initially, a porous amorphous layer of iron oxide forms on the anode surface, consisting of iron(II) hydroxide [Fe(OH)_2_] and iron(III) oxide-hydroxides [FeO(OH), Fe(OH)_3_], along with other metal ions from the solution. However, this layer does not effectively prevent the dissolution of the underlying iron. Over time, further oxidation of the iron oxide-hydroxide leads to the development of a thick, dense black layer containing α-FeO(OH), γ-FeO(OH), and Fe^2+^(Fe^3+^)_2_O_4_, due to reduced oxygen availability at the interface where iron ions dissolve. This behavior, observed at high applied potentials, results in the formation of magnetite Fe^2+^(Fe^3+^)_2_O_4_ particles. The resultant thick black iron oxide layer, exceeding 150 μm in thickness, serves as a passivation barrier that obstructs ion and electron transport, requiring higher electrical potentials to sustain a constant current density and thereby decreasing Faradaic efficiency. Reversing the polarity of the anode to the cathode disrupts this passivating layer through the evolution of hydrogen gas (H_2_) at the interface, thereby restoring low-energy consumption operation by cleaning the surface.

The effectiveness of EC can vary significantly depending on the specific pollutants and experimental conditions as well as the type of electrode material used. For instance, Gong et al. reported that ferric oxide exhibits a higher adsorption capacity compared to aluminum oxide, resulting in superior performance of Fe electrodes over Al electrodes for chemical oxygen demand (COD) and organic compound removal.^37^ Otherwise, under optimal EC conditions applied as a secondary treatment for slaughterhouse wastewater following an anaerobic process, aluminum electrodes demonstrated greater efficiency in COD removal, whereas iron electrodes were more effective in removing nitrate (NO_3_^−^) and turbidity.^41^ Additionally, Fe anodes facilitated faster biofilm formation, thereby reducing the start-up period and improving the performance of a biofilm reactor in subsequent filtration stages.^42^ As illustrated in Figures 4D and 4E, the pH of the wastewater is a critical parameter influencing the removal performance of each electrode during the EC process.^40^ Iron and aluminum hydroxides are generated at specific pH levels, facilitating pollutant removal through precipitation, adsorption, and sweep coagulation mechanisms. In experiments using Fe electrodes, both COD and dye removal efficiencies improved when the wastewater pH was adjusted to 8.5 (COD: 54%, dye: 93%). However, further increasing the pH to 10 resulted in decreased removal efficiencies (COD: 30%, dye: 87%). Nevertheless, for Al electrodes, increasing the pH from 4 to 6 resulted in only a slight improvement in removal efficiencies, with COD and dye removals reaching 38% and 61%, respectively, at pH 10. Based on these observations, further experiments were conducted at pH 8.5 for the Fe electrode and pH 6 for the Al electrode. This indicates that the removal efficiency can fluctuate significantly depending on the specific conditions and electrode materials used.

#### Applied current

**Figure 5:**
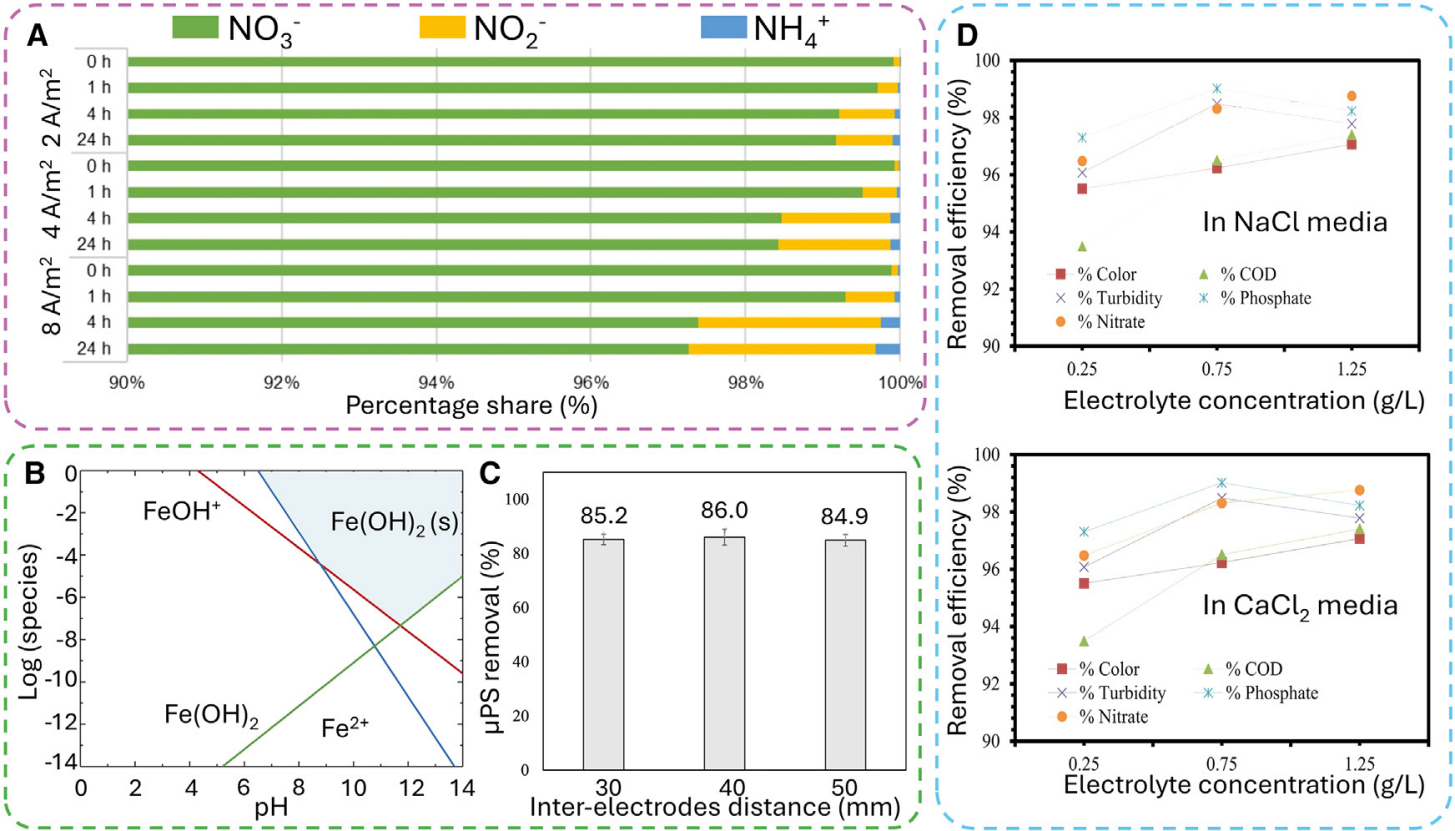
The effect of input varieties to EC performance (A) Percentage of nitrogen forms in influent and treated drainage water by EC system in different current densities.^50^ (B) The distribution of Fe^2+^ species in the aqueous solution at different pH values.^51^ (C) The influence of interelectrode gap on micro polystyrene (μPS) removal performance.^52^ (D) Effect of NaCl and CaCl_2_ electrolytes on removal efficiency.^52^

Current density is a crucial parameter in the EC process, as it influences the rate at which coagulants are dosed and bubble generation, and the size and development of flocs, all of which impact the overall efficiency of the treatment.^43^^,^^44^ As current density increases, the rate of anodic dissolution of sacrificial electrodes also rises, leading to the formation of more hydroxide flocs and thereby enhancing contaminant removal efficiency. It has been observed that the efficiency of effluent removal improves with increasing current density due to the following reasons.^45^ (1) The amount of released metal cations in anode and hydroxide anions in cathode increase, facilitating the sedimentation of contaminants. (2) The rate of bubble generation increases while the bubble size decreases, resulting in enhanced flotation. According to Faraday’s law, an increase in current density and electrolysis duration could lead to a higher anode dissolution rate, thereby increasing the amount of coagulant released from the anode.^46^ Usually, EC shows excellent ability in removing various contaminants, from polar to nonpolar, from organic to inorganic.^47^^,^^48^^,^^49^ However, EC is not effective for treating compounds possessing multiple oxidation states. For example, though the concentration of NO_3_^−^ was effectively decreased due to the reduction reaction, the total nitrogen remained almost unchanged because the partially induced NO_2_^−^ and NH_4_^+^ contributed to maintaining the overall nitrogen levels (Figure 5A).^50^

In conclusion, at low currents, only electrode dissolution occurs, but at high currents, the improvement in pollutant removal efficiency does not increase proportionally with the applied current.^53^ Although efficiency continues to rise with higher current, the rate of increase may become marginal beyond a certain threshold.^54^ This is due to the saturated flocs could hinder the binding of ion hydroxides to pollutants.^55^ Consequently, while applying higher currents does not necessarily enhance decontamination efficiency, it leads to increased energy consumption and, therefore, higher operational costs.^56^ For economic perspective, the hybrid electrochemical process should be optimized to operate at a limiting current density.^57^ Additionally, excessively high currents can lead to competitive electrode reactions and unwanted parasitic reactions, such as oxygen evolution.^58^

#### pH

The pH of the electrolyte is a critical factor in the EC process, as it influences EC performance through its effects on the electrochemical reactions occurring at the anode and cathode.^59^ Besides the intrinsic pH of the electrolyte, the current density could change the local pH during the EC process due to the reduction of water, leading to a rise in pH.^60^ The pH determines adsorption sites and modifies the concentration of positively charged species such as M^n+^, MOH^(*n*−1)+^, and M(OH)_2_^(*n*−2)+^ (MOH represents the hydroxide of a metal cation (M) with an oxidation state of n), etc., which are preferentially adsorbed. The mixing of oxyhydroxides and oxides with varying structures enhances the surface area and porosity of the flocs.^61^ During the EC process, the primary mechanisms of pollutant removal at different pH levels are as follows: (1) charge neutralization at lower pH, and (2) adsorption and sweep flocculation from pH 5.0 to 8.0. Studies have indicated that aluminum-based ions formed within the pH range of 4–9 exhibit a more positively charged surface, making them particularly effective for adsorption, electrochemical neutralization, and net-catching reactions.^62^ However, the coagulation effect diminishes sharply when the pH exceeds 10, and at very low pH values, coagulation becomes negligible.^63^ In the EC system utilizing Fe electrodes, the reactions across various pH levels show similarities to those observed with Al electrodes.^64^ At acidic pH, the predominant species (Fe^2+^/Fe^3+^) are monomeric hydroxo-metallic cations. In neutral conditions, both polymeric hydroxo-metallic cations (FeOH^+^, Fe(OH)_2_^+^, Fe(OH)^2+^, and Fe_2_(OH)_2_^4+^) and metal hydroxide precipitates are present (Figure 5B).^51^ As the pH increases, the formation of ferric oxyhydroxide complexes like Fe(OH)_4_^-^ occurs, which reduces the amount of insoluble hydroxides and increases soluble hydroxides, leading to weaker coagulation and a decrease in removal efficiency.^65^ Consequently, the highest removal efficiency for effluents in the EC system with Fe electrodes is achieved at neutral pH.

To get insights into the mechanism of the EC process, the first-order or second-order pseudo can be used to investigate the interaction between hydroxo-metallic cations and contaminants in the effluent.^66^ Flocculation tends to occur more effectively at lower pH levels, whereas adsorption is more pronounced at higher pH levels.^67^ Typically, as pH increases toward alkalinity, due to the formation of excess hydroxyl ions from the reduction of water at the cathode,^68^ or partial replacement of hydroxyl ions by other anions,^69^ pollutant removal efficiency improves. Conversely, under acidic conditions, the removal efficiency is significantly reduced.^70^ This is because, at low pH, charge neutralization is primarily achieved by monovalent metal ions generated from the anodes.

Additionally, the influence of pH to EC performance can be explained via charging numbers of ions, since the ability of monovalent ions to form coagulation is lower than that of multivalent ions.^71^ The net surface charge of oxyhydroxide particles across different pH levels is determined using zeta potential values.^61^ Above the point of zero charge (pH_pzc_), the surface of oxyhydroxide particles is negatively charged, facilitating the removal of positive ions through both chemical and electrostatic interactions. Below the pH_pzc_, the surface becomes positively charged, which reduces the adsorption of negative pollutants.^72^ Therefore, determining pH_pzc_ and controlling the optimal pH value for specific contaminants during the EC process are important in achieving maximum removal efficiency (Table 2).

**Table 2:** The optimal pH values of different effluents with specific electrode materials in the EC system

Pollutants	Electrode materials	pH range	Optimal pH value	Removal efficiency	Reference
Young landfill leachate	Al	4–8	7.35	83.56% of COD, 73.12% of TSS, 85.58% of TOC	Kundu et al.^73^
Tannery wastewater	Fe	5.5–8.5	5.6	99% of chromium	Aguilar-Ascón et al.^74^
Methylene blue (MB)	Al	3–11	7	97.81% of MB	Hasnaoui et al.^38^
Ni and Cr	Brass	2–10	6–9	99.9% of Ni, 99.38% of Cr(VI)	Patel et al.^75^
Direct violet-35 dye	Al	3–9	7.2	>98% of dye	Sharma et al.^76^
Textile industrial effluent	Al	2–12	7–8	91% of TDS, 79% of COD	Zafar et al.^77^

#### Inert-electrode distance

Optimizing the electrode distance shows a good strategy to enhance EC performance. As known, the charge resistance is proportionally increased with the electrode distance increasing.^78^ As the distance between the anode and cathode increases, both ohmic losses and resistance to ion release increase, adversely affecting mass transfer and charge transfer kinetics.^19^ As a result, it is necessary to increase the applied current to effectively form ions for the coagulation process, which leads to a rise in power consumption. Otherwise, the shorter distance of electrode spacing can favor charge transfer and reduce energy consumption, the high risk of passivation and clogging can prevent electrochemical reactions in the EC cell, leading to disruption of the process. Therefore, optimizing the interelectrode distance is crucial for maximizing pollutant removal efficiency while minimizing power consumption (Figure 5C).^52^

#### Electrolysis time

The reaction time significantly influences the amount of coagulant dissolved from the electrodes, thus determining the removal efficiency of contaminants.^79^ As the reaction time increases, the metal ions and hydroxyl ions released from the electrodes form a greater quantity of hydroxide flocs, leading to the adsorption of a higher number of pollutant particles.^80^ Because EC is a rapid process, the optimal reaction time typically ranges between 20 and 30 min. Shorter reaction times may result in insufficient coagulant formation, resulting in a lower amount of precipitates, with a considerable portion of contaminants remaining in soluble form and consequently lower removal efficiencies.^81^ Additionally, some resistant pollutants may require longer reaction times for complete degradation. Insufficient reaction time may prevent the complete mineralization of particularly resistant organic matter. Conversely, extending the reaction time beyond the optimal range yields negligible improvements in removal performance and instead increases energy and electrode consumption, thereby raising operational costs.^82^ Hence, investigating the optimal reaction time is essential to optimize the removal performance and cost operation.

#### Supporting electrolytes

The electrolytes used in EC systems can offer some parasitic benefits, such as reducing the passivation layer, aiding in disinfection, and promoting complex formation. For example, Kassahun et al. demonstrated that using NaCl or CaCl_2_ can effectively reduce passivation formation, while electrochemically generated chlorine is effective for water disinfection (Figure 5D).^83^ Compared to NaCl, CaCl_2_ is generally more effective due to its higher electrical conductivity. This increased conductivity arises from the intrinsic properties of CaCl_2_ to provide twice the number of positive charge carriers (Ca^2+^) compared to NaCl (Na^+^). Consequently, the addition of NaCl and CaCl_2_ can enhance conductivity, reduce power consumption, and improve process efficiency. In another study, Qi et al. examined the influence of Ca^2+^ on the performance of concurrent EC and electro-Fenton (EF) processes in an iron-EC system.^84^ The presence of Ca^2+^ altered solution pH by competing for binding sites on humic acid (HA) molecules, which could enhance the Fe^2+^/Fe^3+^ ratio. X-ray photoelectron spectroscopy analysis revealed that increased Fe^2+^/Fe^3+^ speciation resulted from the competition among Ca^2+^, Fe^2+^, and Fe^3+^ ions for binding sites on HA, promoting the formation of a tribasic complex of Ca^2+^/Fe^2+^/Fe^3+^-HA and facilitating electron transfer from HA molecules to metal oxides. Although studies examining the effects of increased electrolyte temperature are infrequent in scientific literature, the rise in temperature due to electric current passage (Joule effect) is crucial for assessing the technical feasibility of the EC process.^85^

#### Electrode passivation

The significant challenge remaining in EC systems is the formation of passivation layers on the electrodes, which reduces EC performance.^86^ A well-adhered passive oxide layer on the anode surface inhibits charge transfer between the solution and the electrodes, thereby decreasing the rate of anodic dissolution.^87^ Therefore, to restore the effectiveness of the electrodes, breaking the non-conductive metal oxide layers deposited on the electrode surfaces is crucial. As mentioned previously, using supporting electrolytes such as NaCl and CaCl_2_ can reduce the formation of passivation layers. However, their limitations remain, such as the inclusion of chlorides, which can lead to substantial chemical consumption and, consequently, higher operational costs.^88^^,^^89^

In addition to using supporting electrolytes, several strategies have been proposed to mitigate electrode passivation. For instance, employing a power source with variable pulse frequency or alternating current (AC) can help prevent the formation of passivation layers on electrode surfaces.^90^ Additionally, integrating ultrasound energy with EC has been shown to effectively disrupt electrode passivation.^91^ However, this combined approach may also lead to the disintegration of flocs within the solution. Furthermore, designing electrodes with unique morphologies, such as perforated electrodes, can mitigate passivation and promote better liquid flow between electrodes, reducing sluggishness.^92^ Anaerobic conditions also offer a promising solution to reduce passivation on electrode surfaces. For example, Wu et al. conducted EC using iron electrodes in an anaerobic reactor for wastewater treatment.^86^ The anaerobic environment, coupled with low pH, prevents the accumulation of Fe^3+^ and the formation of suspended sludge during Fe^2+^ release. This approach accelerates Fe^2+^ dissolution and prevents the electrodes from becoming covered with biofilm and accumulated Fe^3+^, effectively addressing the passivation issue.

#### Types of electricity sources

In EC systems, a direct current (DC) power supply is a commonly used electricity source. However, as mentioned earlier, using the DC source in EC systems contributes to the formation of passivation layers, which reduces charge transfer between the electrode and the electrolyte, as well as the dissolution of metal ions. While an AC power supply can mitigate the formation of passivation layers, using AC in EC systems remains some limitations.^93^ These include disrupting the consistency of reactions and interrupting electrode passivation.^28^ Moreover, AC may result in higher energy consumption because ions are frequently redirected without effectively contributing to necessary reactions. Therefore, choosing DC or AC as a power supply in specific EC systems should be carefully considered.

On the other hand, the EC system depends strictly on an external electricity supply that could face challenges in remote or offshore areas lacking electrical infrastructure. With the significant development of sustainable energy technology, there are numerous research that develop self-powered or renewable-driven EC systems.^94^^,^^95^ For instance, Kim et al. created an innovative arsenate removal system that combines EC with an iron-air fuel cell, enabling wastewater treatment in remote areas without the need for an external power supply.^27^ This system is particularly beneficial for regions where electricity access is limited. Similarly, Du et al. explored the implementation of a natural solar energy source for waste treatment, aligning with sustainability and cost-reduction objectives.^96^ This solar-powered EC system was effective in producing large flocs at low current density, and benefited from a gravity-driven membrane bioreactor that supports the growth of beneficial microorganisms, such as biofilms on membrane surfaces. The triboelectric nanogenerator (TENG), an emerging renewable energy technology, shows promise for converting mechanical energy from environmental sources into electrical power, offering a potential alternative electricity source for EC systems. For instance, Zhou et al. developed a self-powered electrochemical system integrating an EC reactor with TENGs, which converted water wave energy into AC output.^97^ This system not only enhances the removal efficiency of organic pollutants and facilitates the separation of water-oil emulsions but also mitigates electrode passivation effects with low-frequency AC generated by the TENG. This study paves the way for treating oil-water waste from oil spills in surface seawater.

## Application of EC process

Wastewater is often contaminated with a diverse array of both organic and inorganic pollutants, and EC has shown outstanding performance for its effectiveness in addressing this broad spectrum of contaminants. The process is widely employed in the treatment of various effluents, including those from industrial, agricultural, and domestic sources. Its popularity is attributed to its operational simplicity, environmental friendliness, and adaptability, making it a versatile solution for managing wastewater across multiple sectors. Comprehensive details regarding the experimental conditions required to achieve optimal removal efficiency for treating various wastewater sources are presented in Table 3.

**Table 3:** Review of studies on waste removal from water using EC method

No.	Influent	Cell design	Assisted method (pre- or post- or co-EC setting)	Experimental condition	Anode/cathode and removal rate (%)	Applied voltage or current	Working time	Electrical energy consumption	Reference
1.	Trace metal	Parallel (electrode surface of 54 cm^2^)	None	pH = 8.5	Al//Al (Cd = 82%, Pb = 84%) Fe//Fe (Cd = 90%, Pb = 85%)	j = 9.2 mA/cm^2^	2 min	N/A	Heffron et al.^33^
2.	Chromium	Monopolar arrangement (10 equidistant iron electrodes of 13.5 cm × 5.5 cm)	None	pH = 4-8 T = 25°C	Fe//Fe (Cr removal = 98.76%)	15 V	180 min	14.3 KWh/m^3^	El-Gawad et al.^98^
3	Gold	Parallel (iron electrode of 10 cm × 10 cm × 1 mm and titanium electrode of 10 cm × 10 cm × 0.5 mm)	None	pH = 6.5	Fe//Ti (Au removal = 100%)	0.5 V	80 min	N/A	Dai et al.^99^
4	Industrial swine slaughterhouse wastewater	10 aluminum electrodes (16.0 cm × 4.0 cm × 0.55 cm, 99.7% purity) as the anodes and cathodes in continuous operating mode	None	N/A	Al//Al (Total organic carbon removal = 72.7%)	25 mA/cm^2^	N/A	19.80 KWh/m^3^	Sandoval et al.^100^
5	Carwash wastewater	5 tubes of Al electrode (75 mm × 8 mm) and 5 tubes of Fe electrode (75 mm × 8 mm)	None	N/A	Al//Fe (COD removal = 89%)	2 mA/cm^2^	8.6 min	7.3 kWh/m^3^	Zivari-Moshfegh et al.^101^
6	Polystyrene microplastics	An electrode spacing of 1 cm	None	pH = 7	Al//Al (removal efficiency = 93.06%)	0.2 A	60 min	N/A	Subair et al.^102^
7	Nickel and copper ions	Parallel (iron electrodes of 12 cm × 7 cm)	Adsorption by waste tea residue (pre-EC)	T = 25 ± 1°C pH = 4	Fe//Fe (Ni = 99.99% and Cu = 100%)	j = 1.19 mA/cm^2^	30 min	0.8125 KWh/m^3^	Jean Claude et al.^103^
8	Phosphate	Monopolar arrangement (iron electrodes of 16 cm × 12 cm)	Biofilm process (pre-EC)	T = 26 ± 1°C pH = 4	Fe//Fe (phosphate removal = 90.24%)	2.1 mA/cm^2^	34 min	N/A	Zeng et al.^104^
9	Dairy manure slurry	A cylindrical anode and a cylindrical cathode with 1 cm in distance	Chemical coagulation (pre-EC)	pH = 7	Al steel//stainless steel	1 A	30 min	N/A	Meetiyagoda et al.^105^
10	Copper	Monopolar arrangement (electrode surface of 36 cm^2^)	Flotation (co-EC)	pH = 7 T = 20°C	Al//Al (Cu removal = 100%)	2.4 mA/cm^2^	30 min	N/A	Kashi^106^
11	Imidacloprid	Parallel (3 anodes and 3 cathodes with a total active surface area of 98 cm^2^)	Ultrasonication (co-EC)	pH = 6.9 T = 20 ± 0.5°C	Al//Al system (removal ∼100%) Cu//Cu system (removal = 87.04%) Fe//Fe system (removal = 96.51%)	20 A	20 min	76.11 KWh/m^3^	Hashim et al.^107^
12	Beet sugar	Tubular screen roll anode and two cathodes	Electromagnetic assist (co-EC)	N/A	Fe//Fe (removal = 97.7%)	3.13 A/m^2^	65 min	2.569 KWh/m^3^	Fadali et al.^108^
13	HA and metal ions	Parallel (Ti/Ti_4_O_7_ electrode of 10 cm × 7 cm and Al electrode of 10 cm × 7 cm)	Electrochemical oxidation (co-EC)	T = 23 ± 1°C	Ti/Ti_4_O_7_//Al (HA removal = 84.2%)	20 mA/cm^2^	3 h	N/A	Hu et al.^109^
14	Direct Red 89 (DR89)	Electrode size (10 cm × 10 cm × 2 mm)	Peroxydisulfate and hydrogen peroxide activation (co-EC)	pH = 3	Al//Fe (DR89 removal = 85.26%)	20 mA/cm^2^	15 min	167.1 kWh/kg	Karimi et al.^110^
15	Inorganic wastewater	Monopolar arrangement (electrode surface of 40 cm^2^)	Electrooxidation (Ti/Pt anode//stainless steel cathode) (post-EC)	pH = 4.67 Room temperature	Al//Fe system (removal rate: COD = 96.1%; color = 97.5%; turbidity = 90.9%; TOC = 98%)	2.6 A	31.67 min	0.01 kWh/kg for COD; 0.008 kWh/kg for color; 0.062 kWh/kg for turbidity; 0.079 kWh/kg for TOC	Mousazadeh et al.^111^
16	Palm oil	Electrode configuration of 2 anodes, 2 cathodes, and 2 bipolar. Electrode size (33 cm × 4 cm × 3 mm)	Ultrafiltration (post-EC)	N/A	Al//Al Removal rates of TDS, TSS, COD, and BOD are 59.1%, 99.9%, 96.8%, and 96%	132.74 An m- 2	60 min	6.20 kWh/m^3^	Aryanti et al.^112^
17	Organic matter from landfill leachate	Parallel (electrodes of 18 cm × 2.5 cm)	Treatment wetland (post-EC)	–	Al//Fe (COD removal = 79.4 ± 0.16%)	20 V	–	N/A	Pinedo-Hernández et al.^113^
18	Sulfonated humic acid (SHA)	2 iron anodes (15 cm × 12 cm × 0.3 cm) and 2 graphite cathodes (15 cm × 12 cm × 1 cm) with 1.5 cm between each electrode	Ultrafiltration (post-EC)	pH = 5	Fe//C (SHA removal = 95%)	10 mA/cm^2^	7 min	N/A	Han et al.^114^
19	Chloride ions	Electrode size (10 cm × 11 cm) and electrode distance of 1 cm	Electrodialysis (post-EC)	N/A	Al//C (Cl^−^ removal = 83%)	N/A	36 min	7.7 kWh/m^3^	Ebrahimi Gardeshi et al.^115^
20	Landfill leachates	Monopolar electric connection (5 anodes and 5 cathodes with 168 cm^2^ in area and electrode distance is 1 cm)	Membrane bioreactor + nanofiltration (post-EC)	pH = 6	Fe//Fe (the total organic carbon removal = 65%)	3 mA/cm^2^	3 h	21 kWh/m^3^	Mostefaoui et al.^116^
21	Phosphorous recovery	0.5 cm of inter-electrode distance	Flotation (post-EC)	pH = 8	Mg//C (98% of phosphorus harvesting efficiency)	40.78 mA/cm^2^	15 min	4.03 kWh/kg	Nageshwari et al.^19^

Industries generate substantial quantities of pollutants, including refractory inorganic and organic compounds, which contribute to toxicity, discoloration, and odor issues in aquatic environments and ecosystems. The effluents from various industrial processes are diverse and complex, often containing solid particles of varying sizes, which complicates their removal. Consequently, it is essential to treat these wastewater effluents before discharging them into surface water bodies or sanitary sewers.

Inorganic wastewater primarily consists of anions and cations, which can be effectively removed using EC. Chromium is essential for carbohydrate metabolism at trace levels, but elevated concentrations can be harmful.^117^ The discharge of chromium-containing industrial effluents has become a global concern, affecting both human health and ecosystems.^118^ Among chromium species, hexavalent chromium (Cr(VI)) ions are particularly hazardous, posing significant environmental and public health risks. Typically, *ex situ* treatment methods for chromium removal involve CC using ferrous sulfate (FeSO_4_), which reduces Cr(VI) to Cr(III) through the oxidation of Fe^2+^ to Fe^3+^.^119^ The ferric hydroxides formed during this process possess a large surface area and act as sorption agents for chromium, enabling its removal from water through subsequent sedimentation and filtration. However, traditional methods such as aluminum or ferric coagulation and lime softening are ineffective for removing Cr(VI) due to the high solubility of Cr_2_O_7_^2−^ and dichromate ions. In contrast, EC offers an alternative approach, where electrodes made of iron, aluminum, or alloys reduce Cr(VI) to Cr(III), followed by precipitation as Cr(OH)_3_ via sweep coagulation.^120^ EC is also a highly effective post-treatment technology for the removal of anions such as cyanide (CN⁻) and fluoride (F⁻), offering exceptional capacity at a relatively low cost.^121^^,^^122^ In the steel industry, the generation of CN⁻ in industrial wastewater is substantial due to high manufacturing rates. Globally, up to 90% of steel industry wastewater is discharged without adequate treatment, leading to the release of cyanide-contaminated effluents into natural water bodies. Without proper treatment, CN⁻-based contaminants pose significant health risks, including both chronic and acute toxicity.^123^ To address this problem, EC, supported by ozonation, has demonstrated an exceptional cyanide removal efficiency of 99.8%.^124^ In contrast, when EC or ozonation is used alone, the removal efficiency for cyanide is significantly lower. This hybrid approach enhances the overall treatment process, highlighting the importance of integrated strategies for managing hazardous contaminants in industrial wastewater.

EC is a robust technique for degrading highly resistant organic compounds, demonstrating outstanding performance in colloidal removal.^125^^,^^126^ The formation of colloidal particles is a critical factor in coagulating organic compounds, as these particles enhance the aggregation and removal of contaminants from wastewater. For instance, organic compounds such as citrate, ethylenediaminetetraacetic acid (EDTA), tartrate, and HA can form stable, water-soluble chelates with heavy metals, making these complexes more resistant to removal compared to free metal ions.^93^^,^^127^ The EC process has demonstrated exceptional effectiveness in treating wastewater contaminated with copper-EDTA complexes, achieving a remarkable copper removal efficiency of 99.85%.^128^ This high efficiency underscores the capability of EC to tackle challenging contaminants that form stable chelates with heavy metals.

Landfill leachate is a complex and highly toxic wastewater generated when water from rainfall, groundwater, or other sources infiltrates solid waste landfill sites.^129^ This leachate contains a heterogeneous mixture of pollutants, both organic and inorganic, many of which are non-biodegradable.^130^ The composition of leachate can vary significantly, with its biochemical oxygen demand (BOD) to COD ratio changing over time. Initially, the BOD/COD ratio is high due to the presence of biodegradable pollutants, but as these pollutants are consumed by bacteria, the ratio decreases while nonbiodegradable chemicals persist, maintaining high COD levels. To mitigate the environmental hazards posed by leachate, effective treatment is crucial. Various treatment methods are employed, including biological, physical, and chemical processes, as well as hybrid techniques. Biological treatments encompass both aerobic and anaerobic processes, while physical and chemical methods include sedimentation, flotation, coagulation, flocculation, adsorption, and chemical oxidation.^131^ Despite these approaches, substantial concentrations of COD and suspended solids (SS) often remain, posing challenges to treatment. Among various treatment approaches, EC is particularly effective for treating landfill leachate due to the coagulation and flocculation of particles, facilitating their removal through sedimentation or subsequent treatment stages. However, to enhance removal effectiveness, EC must be combined with other techniques, such as membrane processes, coagulation and precipitation, and adsorption.

In addition to effectively removing contaminants for the safe and sustainable utilization of water after treatment, the EC system must ensure that residual concentrations of coagulant metals comply with domestic or drinking water standards. Groundwater, often considered a safe source of drinking water in urban areas, has been contaminated due to factors such as weathering and erosion of mineral rocks and the improper disposal of livestock waste and fertilizers.^132^ These artificial and natural contaminants lead to significant pollution of groundwater and surface water resources, resulting in a scarcity of safe potable water and increased adverse effects on human health.^133^ To address these issues and ensure water safety, purification of drinking water is essential. EC has demonstrated effectiveness in reducing contaminant levels to below regulatory thresholds for drinking water.^134^^,^^135^ For example, Cu concentrations above allowable limits in drinking water can adversely affect human health and aquatic ecosystems. Giti Kashi demonstrated that coupling EC with flotation techniques, such as air stones and aerated jet pumps, can achieve 100% removal of Cu^2^⁺ from urban drinking water.^106^ This strategy paves the cost-affordable way to remove complete contaminants with simple equipment.

Although EC is effective in eliminating contaminants from water, challenges related to coagulant residuals as secondary pollutants require further investigation. Aluminum (Al) and iron (Fe) are regulated for their secondary maximum contaminant levels in drinking water, set at 0.2 mg/L and 0.3 mg/L, respectively. Although these standards are not mandatory by the US Environmental Protection Agency, they are critical for the successful implementation of water treatment technologies. Excessive Fe concentrations can discolor drinking water and lead to clogs in plumbing, while chronic ingestion of Al may pose neurotoxic risks.^136^^,^^137^ Thus, maximizing effluent removal and minimizing the concentration of Al and Fe residues in drinking water should be aligned to ensure both effective and safe water treatment.

## Integrated EC systems

Previous studies have demonstrated that relying solely on EC is often insufficient for the efficient treatment of diverse wastewater types. By integrating EC with methods like adsorption, ozonation, or membrane filtration, the combined process can achieve higher pollutant removal rates, improved treatment stability, reduced reaction time, and more comprehensive water quality management compared to single-one techniques. Coupling EC with other methods can be categorized into four primary architectures: biological-, physical-, chemical-, and electrochemical systems. In the following sections, we will discuss the role of combined methods in pre- or post-EC positions for addressing a broad range of contaminants.

### Integrated biological-EC system

Treating effluents with high COD concentrations using a single-step EC process presents significant challenges because of incomplete removal.^138^ Specifically, EC struggles to handle wastewater with high organic loads (COD >10,000 mgO_2_/L) due to the excessive coagulant doses required. In order to achieve these doses, higher current density and/or longer treatment time are required, which leads to an increase in operational costs. Therefore, some suitable approaches such as wastewater dilution before EC treatment, or integrating EC with a biological approach, could be promising strategies for treating effluent with high COD concentration. For example, as an effective pre-treatment method, the removal efficiency of COD and total phenols was significantly enhanced, which corresponds with increased biochemical methane production.^139^

**Figure 6:**
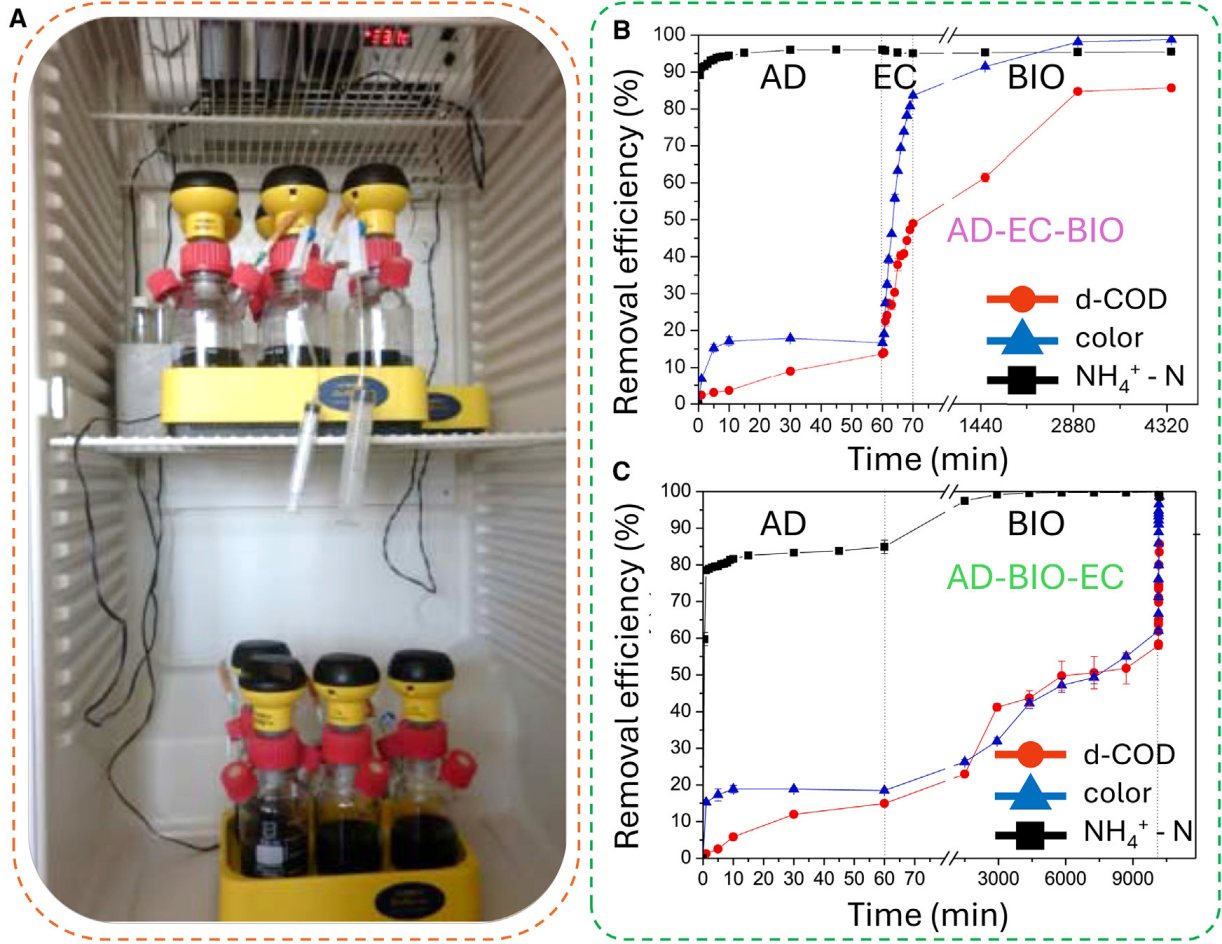
The integrated biological-EC system (A) The anaerobic batch reactor.^140^ (B and C) Overall performance of the hybrid systems on ΝΗ^4+^-Ν, color, and d-COD removal efficiencies: (B) AD-EC-BIO and (C) AD-BIO-EC (AD: after adsorption with zeolite; EC: after electrocoagulation; BIO: after biological treatment with plastic tubes).^141^

In combined EC systems, as the post-treatment method, EC can enhance overall removal performance by tackling residuals that cannot be removed by the preceding method. For example, combining EC with anaerobic biological processes addressed the limitations of anaerobic digestion by removing non-biodegradable organic effluents through co-precipitation with metal hydroxides and/or adsorption onto these hydroxides (Figure 6A).^140^

In hybrid systems, the order of each method influences the overall performance of the system. For example, a three-stage pilot study employing adsorption as a pre-treatment step in sequential treatment scenarios (AD-EC-BIO and AD-BIO-EC) highlighted the importance of the order of each method in treating raw sanitary landfill leachate (Figures 6B and 6C).^141^ The AD-EC-BIO system effectively removed colored compounds (anionic aromatic and aliphatic compounds) and d-COD (containing HA) due to the electrostatic attraction between these compounds and the positively charged Al hydroxides formed during coagulation. In conclusion, integrating with biological methods, EC shows promising with high effectiveness in pre- or post-treatment methods, achieving nearly complete removal of contaminants.

### Integrated physical-EC system

The flotation process, utilizing air bubbles to float contaminants without the addition of coagulation agents, can significantly enhance the performance of EC both economically and environmentally.^142^ This hybrid approach can be utilized for treating liquid effluents from anaerobic digestion, which often have high levels of BOD, COD, and nutrients. For instance, integrating external aeration with EC can boost performance significantly; while EC alone achieved a 15% reduction in COD, the support of aeration increased this reduction to over 40%.^143^ Using by-product gases from anaerobic digestion, such as hydrogen sulfide (H_2_S), in the flotation process can provide benefits by enhancing flotation and reducing the amount of H_2_S, which is the primary cause of damage to gas-handling equipment. For example, Liu et al. developed a dual-purpose approach that simultaneously addresses water reclamation and biogas purification by precipitating H_2_S as sulfate-based compounds using Fe^3+^ from the EC process.^144^ This approach also controls the high pH environment of the electrolyte, improving the flotation of AD effluent and achieving further reductions in turbidity and COD by 60% and 10%, respectively.

**Figure 7:**
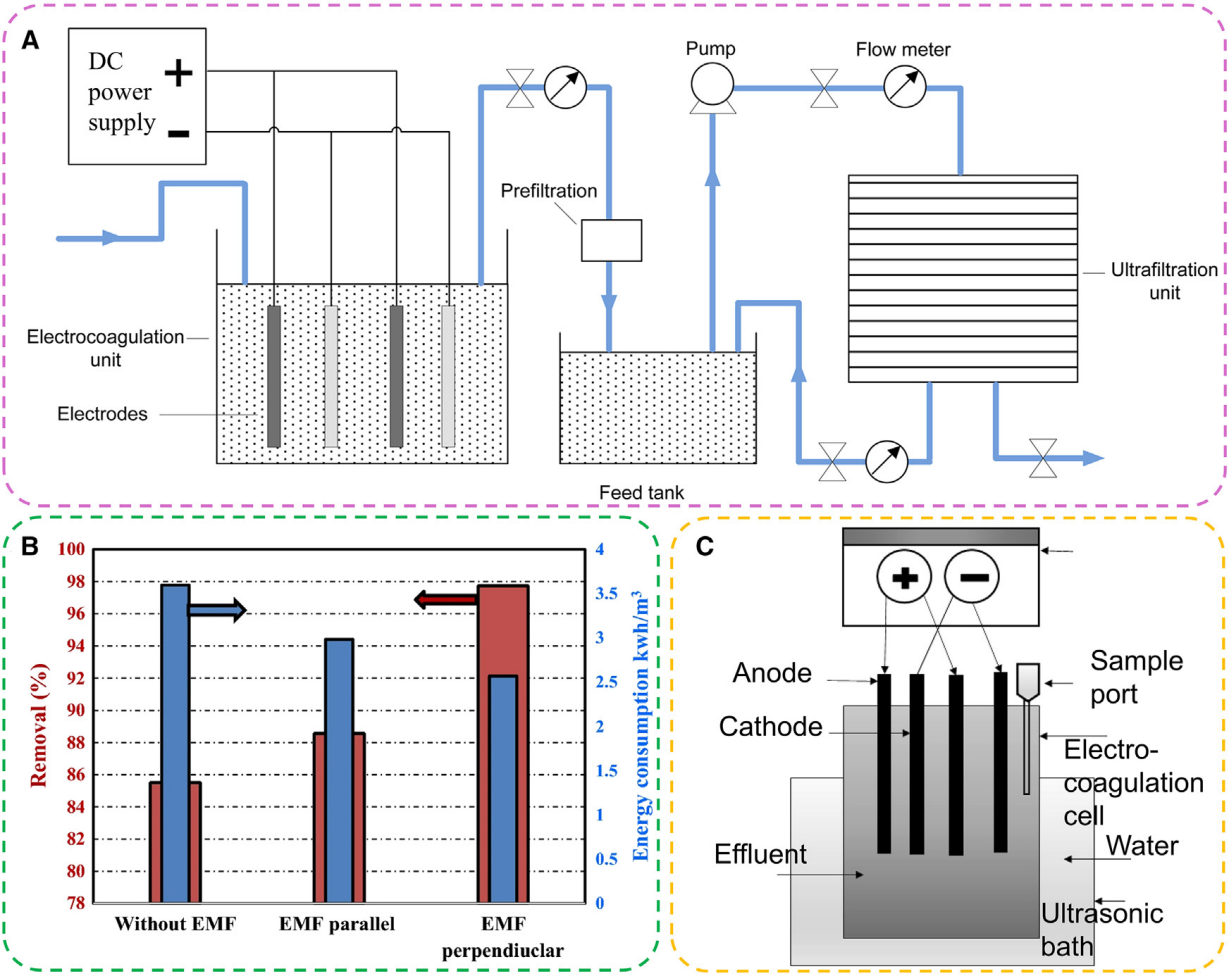
The integrated physical-EC system (A) Schematic illustrator of the hybrid EC-ultrafiltration treatment system.^114^ (B) Energy consumption (in kWh/m^3^) and % color removal for the two positions of the electromagnetic field (EMF) compared to the experiment without EMF.^108^ (C) Schematic diagram of sono-EC.^147^

Among physical approaches, adsorption is one of the most popular methods for wastewater treatment. Therefore, combining EC with adsorption, without the use of chemicals or activating agents, can demonstrate exceptional performance in removing effluents.^103^ The integration of EC with membrane filtration treatment is also one of the most promising strategies gaining attention due to its high selectivity and surface area, which enhance the separation and treatment processes.^145^ The big size of floc generated from EC is typically easy to separate through filtration. Therefore, coupling EC with ultrafiltration membranes can significantly improve removal efficiency and minimize fouling of membranes (Figure 7A).^114^ For example, using a hybrid EC-ultrafiltration system achieved a COD removal efficiency of 99.18%, compared to 89.6% with EC alone.^146^ Thus, the hybrid EC-membrane system, by minimizing fouling and enhancing permeation flux, offers a superior alternative to conventional membranes.

Magnetohydrodynamics (MHD) is the field incorporating the magnetic field and electrically conducted fluid systems. One of the probable actions of the MHD force at the electrode surface is to facilitate the transportation of the hydrogen bubbles from the cathode and the metal ions from the anode. Due to the faster removal of pollutants in the presence of electromagnetic field (EMF) compared to the experiment without the EMF, the energy consumption can be reduced by the addition of EMF (from 3.595 to 2.569 kWh/m^3^) as shown in Figure 7B.^108^ This study has proven to be a promising method for high waste removal with a significantly lower power consumption compared to currently available techniques.

Combining ultrasonication with EC can effectively mitigate electrode passivation through the mechanical effects of ultrasonication cavitation (Figure 7C).^147^ During ultrasonication, acoustic cavitation generates highly reactive oxygen species, such as hydrogen atoms (H⋅) and hydroxyl radicals (OH⋅) from water (H_2_O → H⋅ + OH⋅). These reactive species play a crucial role in the oxidation and reduction of both organic and inorganic molecules present in the water. Enhanced chemical reactivity accelerates the removal rate of contaminants in the hybrid EC-ultrasonication process. Moreover, the mechanical action of cavitation disrupts the passivation layer that forms on the electrode surfaces, maintaining optimal electrode performance and improving overall treatment efficiency.

### Integrated chemical-EC system

**Figure 8:**
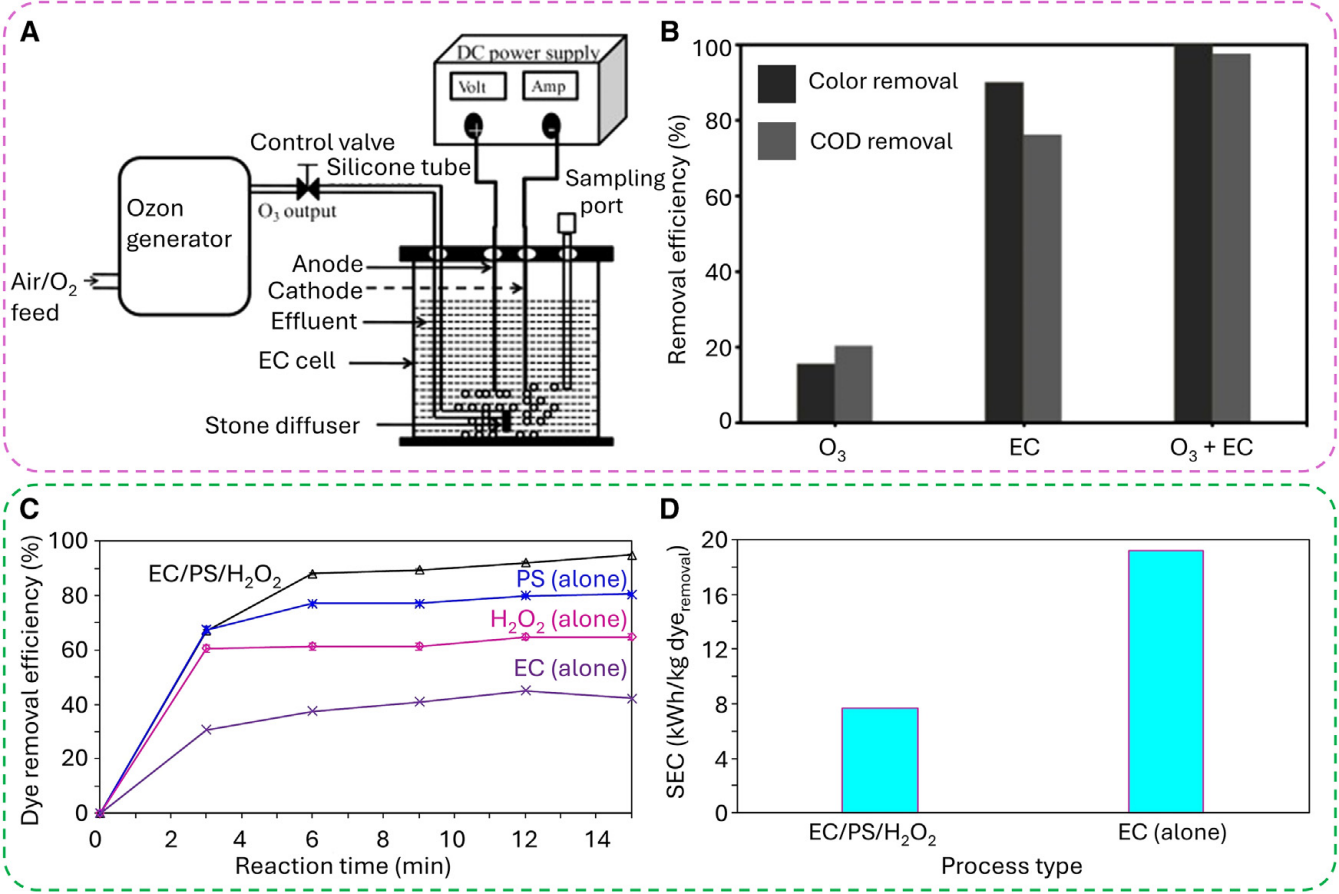
The integrated chemical-EC system (A and B) (A) Experimental setup of ozone-assisted EC and (B) comparison of ozonation, electrocoagulation, and ozone-assisted EC (current density: 3 A/dm^2^, effluent COD concentration: 3,000 ppm, pH = 7, inter-electrode distance: 1.8 cm, electrolysis time: 5 h, and ozone flow rate: 15 L/min and concentration: 2 g/h).^150^ (C and D) (C) Comparison of DR89 dye removal efficiency by different processes and (D) energy consumption for different processes. Experimental condition: electric current = 0.1 A, (PS) = 0.4 mM, (H_2_O_2_) = 0.4 mM, (Na_2_SO_4_) = 2 mM, pH = 7, (DR89) = 100 mg/L, reaction time = 15 min, electrode: Al//Fe.^110^

Several researchers have explored the application of ozone-assisted EC for the treatment of synthetic effluents. Notably, this combined approach enhances the removal efficiency of color and COD. The coupling of EC with ozone has proven highly effective in achieving significant organic and inorganic contaminant removal, while simultaneously reducing energy consumption and minimizing costs.^124^^,^^148^^,^^149^ In the context of distillery wastewater treatment, the integrated ozone-EC method has emerged as an environmentally friendly and cost-efficient approach, achieving nearly complete removal of color (100%) and high COD reduction (95%) (Figure 8A).^150^ Experimental data further revealed that the ozone-assisted EC process achieved superior pollutant removal compared to the individual application of either ozone or EC (Figure 8B). Industrial textile wastewater, which typically exhibits high pH, intense coloration, and extreme salinity, especially following dyeing processes, was effectively treated using the combined EC and ozone approach, offering greater economic benefits compared to the single EC process. Furthermore, ozone-assisted EC was successfully applied for the decolorization of synthetic wastewater containing acid dyes in an innovative single airlift reactor configuration, proving to be more cost-effective than when conducted in separate conventional reactors. For example, the introduction of ozone into the EC system induces a catalytic reaction between Fe^2+^ and O_3_, resulting in the formation of intermediate FeO^2+^ (Equation 5), this ion that subsequently generates hydroxyl radicals (⋅OH) (Equation 6).^151^

(Equation 5) $$O_{3}+{\text{Fe}}^{2+}\;\to \;{\text{FeO}}^{2+}+O_{2}$$

(Equation 6) $${\text{FeO}}^{2+}+H_{2}O\;\to \;{\text{Fe}}^{3+}+\text{HO}\cdot +{\text{HO}}^{-}$$

The presence of radicals such as sulfate (⋅SO_4_^−^) or hydroxyl (⋅OH) generated from oxidants like persulfate (PS) or H_2_O_2_ could be the appealing factor to significantly enhance the efficiency of the EC process. These radicals promote the formation of more gas bubbles, which facilitate flotation, and reduce electrode surface passivation. The integrated EC/PS/H_2_O_2_ process, demonstrated a dye degradation efficiency of 94.82%, outperforming other methods (Figure 8C).^110^ Furthermore, this combined approach consumed 2.49 times less energy compared to conventional EC (Figure 8D).

In another example, Fenton’s reaction utilizes non-complexed Fe^2+^ and H_2_O_2_ to generate hydroxide radicals (⋅OH) (Equation 7). These radicals react rapidly with Fenton’s reagents (Equations 9, 10, and 11) in nanoseconds. This rapid reaction often depletes both oxidants and reagents, prematurely terminating the Fenton cycle and limiting its treatment efficacy. However, under acidic conditions (pH 2–4), soluble Fe^3+^ can be recycled into Fe^2+^ (Equation 8), thereby sustaining the generation of hydroxyl radicals without reagent exhaustion.

(Equation 7) $${\text{Fe}}^{2+}+H_{2}O_{2}\;\to \;{\text{Fe}}^{3+}+\text{HO}\cdot +{\text{OH}}^{-}$$

(Equation 8) $${\text{Fe}}^{3+}+H_{2}O_{2}\;\to \;{\text{Fe}}^{2+}+{\text{HO}}_{2}\cdot +H^{+}$$

(Equation 9) Fe2++14O2+2OH−+12H2O→Fe(OH)3

(Equation 10) $${\text{Fe}}^{2+}+\text{HO}\cdot \;\to \;{\text{Fe}}^{3+}+{\text{OH}}^{-}$$

(Equation 11) $$H_{2}O_{2}+\text{HO}\cdot \;\to \;{\text{HO}}_{2}\cdot +H_{2}O$$

However, despite the higher removal performance of coupled EC-CC systems, the addition of chemical coagulants and the production of denser sludges hinder the affordability of commercial-scale wastewater treatment.

### Integrated electrochemical-EC systems

Over the past two decades, electrochemical advanced oxidation processes (AOPs) have garnered significant attention for their efficacy in degrading a wide array of hazardous and recalcitrant organic compounds.^152^ AOPs operate on the principle of electrochemical generation of hydroxyl radicals, potent and non-selective oxidizing agents. These processes offer several technical advantages, including versatility, ease of operation, automation potential, and minimal chemical consumption. The most widely utilized electrochemical AOPs are electrooxidation (EO), EF, and their various modifications.^153^^,^^154^ The following section will discuss the coupling EC and advanced oxidation methods.

#### Integrated electrooxidation-EC system

EO employs inert electrodes to directly oxidize contaminants on the electrode surface by generating hydroxyl radicals at the anode or by producing oxidants in the solution.^155^^,^^156^ Unlike EC, EO is a more time-intensive process but is highly effective in ensuring the complete removal of pollutants. The performance of EO is strongly influenced by the choice of anode material.^111^ Traditional RuO_2_-based electrodes, long considered the benchmark for EO, face limitations due to their expensive cost and limited elemental abundance. To address these challenges, alternative materials such as tin dioxide (SnO_2_) and titanium dioxide (TiO_2_) have been developed.^157^^,^^158^^,^^159^ However, these alternatives are hindered by issues such as potential chemical leaching and shorter operational lifespans, which limit their commercial viability.^160^^,^^161^

Anodic oxidation can proceed via two primary mechanisms: direct and indirect oxidation. In direct EO, also known as the electrochemical oxygen transfer reaction, pollutants are adsorbed onto the anode surface, where they undergo direct electron transfer and are oxidized on the electrode. On the other hand, in indirect EO, also referred to as mediated anodic oxidation, chemical oxidants are generated *in situ* via anodic oxidation-such as active chlorine or PS, or through cathodic reduction, as in the case of hydrogen peroxide. EO can also produce active chlorine species when chloride ions are present in the system. The production pathways for hydroxyl radicals and active chlorine species are illustrated in Equations 12, 13, 14, and 15.

(Equation 12) $$M+H_{2}O\;\to \;M\left(\cdot \text{OH}\right)+H^{+}+e^{-}$$

(Equation 13) $$2H_{2}O\;\to \;O_{2}+4H^{+}+4e^{-}$$

(Equation 14) $$2{\text{Cl}}^{-}\;\to \;{\text{Cl}}_{2}+2e^{-}$$

(Equation 15) $${\text{Cl}}_{2}+H_{2}O\;\to \;\text{HClO}+{\text{Cl}}^{-}+H^{+}$$

**Figure 9:**
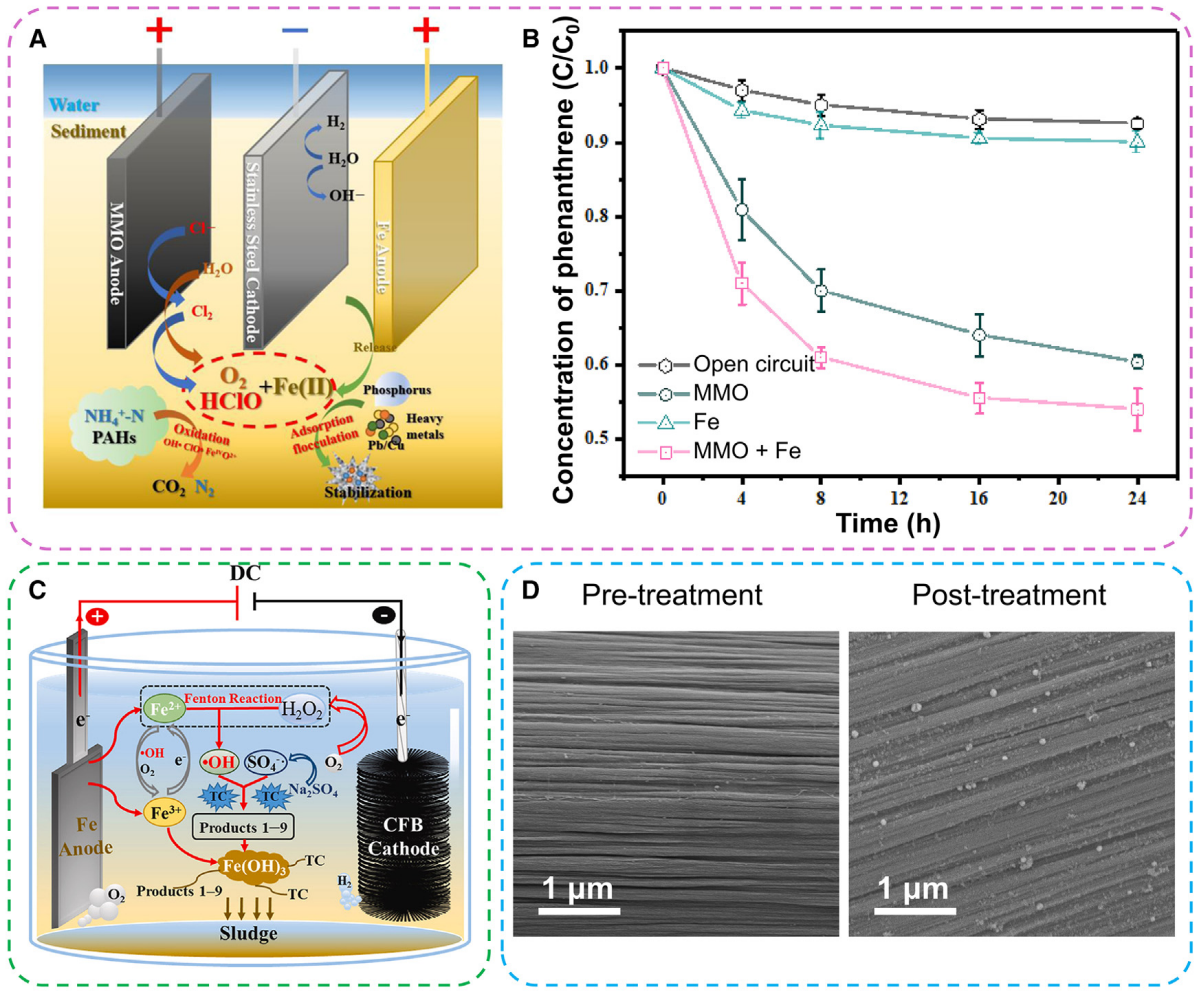
The integrated electrochemical-EC systems (A) Schematic diagram of the EC coupled with electrooxidation for the simultaneous treatment of multiple pollutants in contaminated sediments, and (B) time course of phenanthrene concentrations in the sediment as a result of different treatments. For single mixed metal oxide (MMO) and single Fe anodes, the current density was 20 mA/cm^2^. For dual MMO/Fe anodes, the current density applied to MMO and Fe was 10 mA/cm^2^ and 10 mA/cm^2^, respectively. The initial contaminant concentration was 1.00 ± 0.1 mg/kg).^163^ (C) Schematic diagram of the synchronous degradation and removal mechanism in the Fe-carbon fiber brush (CFB) system. (D) SEM images of CFB cathode before and after use.^164^

Rapid and complete pollutant removal can be achieved by combining EC and EO through either sequential or simultaneous applications.^162^ For instance, Zeng et al. designed a hybrid EC/EO system within a single unit, demonstrating its efficiency in immobilizing phosphorus and heavy metal ions, as well as oxidizing ammonium and toxic organic compounds (Figure 9A).^163^ This integrated EC/EO reactor enables the concurrent treatment of multiple pollutants in sediments by leveraging the synergistic effects of coagulation, driven by *in situ* electrogenerated Fe(II) at the iron anode, and oxidation, driven by reactive oxygen and chlorine species at MMO anode. Compared to systems utilizing either a single Fe anode or a single MMO anode, the dual-anode configuration (MMO/Fe) substantially enhanced the removal efficiency of NH_4_^+^ and P in the overlying water, reduced phenanthrene (Phen) levels in sediments, and improved the stabilization and immobilization of heavy metals such as Pb and Cu. As illustrated in Figure 9B, the combined use of MMO and Fe anodes achieved superior effluent quality compared to the performance of each anode individually. This improvement is likely due to the *in situ* generation of Fe(II) at the Fe anode, which activates reactive oxygen and chlorine species produced at the MMO anode, thereby generating a greater quantity of oxidative species that effectively degrade organic pollutants. Thus, the integration of EC and EO presents one of the most promising electrochemical approaches for the efficient removal of contaminants from wastewater.

#### Integrated Fenton-EC system (peroxi-coagulation process)

The peroxi-electrocoagulation (PC) process combines two prominent electrochemical techniques: the EF process, an advanced oxidation method, and EC, an electrochemical separation process.^165^^,^^166^ In the EF process, ferrous ions (Fe^2+^) and hydrogen peroxide (H_2_O_2_) are generated *in situ* during electrolysis, following the reactions represented in Equations 16 and 17. This simultaneous production of reagents enhances the oxidative capacity of the system, facilitating the degradation of a wide range of organic contaminants.

(Equation 16) $$\text{Fe}\;\to \;{\text{Fe}}^{2+}+2e^{-}$$

(Equation 17) $$O_{2}+2H^{+}+2e^{-}\;\to \;H_{2}O_{2}$$

In electrochemical processes, the applied current density plays a critical role as an electrokinetic regulator, which decides the production rate of hydroxyl radicals (HO⋅), the cathodic generation of H_2_O_2_, and the rate of Fe^3+^ are reduced to Fe^2+^.^167^ However, excessively high current densities can negatively impact pollutant removal efficiency. This may occur because elevated current densities increase the bulk solution’s temperature, leading to a higher concentration of stabilized contaminants, which resists further treatment.^168^^,^^169^ Additionally, parasitic side reactions consume the HO⋅ radicals, further diminishing their availability for contaminant oxidation.^169^ At higher current densities, the abundance of Fe^2+^ and H_2_O_2_ ions in the solution increases, which leads to the reaction of excess Fe^2+^ with HO⋅ radicals, resulting in the formation of additional Fe^3+^ ions (Equation 18). These ferric ions subsequently interact with H_2_O_2_ to generate hydroperoxyl radicals (HO_2_⋅), which are weaker oxidants compared to HO⋅ (Equation 19). Hydroperoxyl radicals can also form through the reaction between H_2_O_2_ and HO⋅ (Equation 20). Furthermore, the dimerization of HO⋅ radicals into H_2_O_2_ is another possible reaction at higher current densities, reducing the overall oxidative capacity of the system (Equation 21).

(Equation 18) $${\text{Fe}}^{2+}+\text{HO}\cdot \;\to \;{\text{Fe}}^{3+}+{\text{HO}}^{-}$$

(Equation 19) $$H_{2}O_{2}+\text{HO}\cdot \;\to \;{\text{HO}}_{2}\cdot +H_{2}O$$

(Equation 20) $${\text{Fe}}^{3+}+H_{2}O_{2}\;\to \;{\text{Fe}}^{2+}+H^{+}+{\text{HO}}_{2}\cdot$$

(Equation 21) $$2\text{HO}\cdot \;\to \;H_{2}O_{2}$$

In the electro-Fenton-electrocoagulation (EF-EC) process, the performance of H_2_O_2_ production is determined by the two-electron reduction of oxygen (O_2_), which diffuses from the solution and adsorbs onto the cathode surface.^170^^,^^171^ Consequently, a cathode with a large specific surface area is more conducive to O_2_ adsorption and enhanced H_2_O_2_ generation. For example, Feng et al. developed an EF-EC system utilizing a durable and stable carbon fiber brush (CFB) cathode, which provides a substantial surface area that optimizes H_2_O_2_ production.^164^ As depicted in Figure 9C, the surface of the CFB cathode appeared clean and free of impurities before use. Post-operation, the carbon fiber filaments were partially covered with flocs, but no significant alteration in the surface morphology or structure was observed (Figure 9D), indicating the viability of CFB cathodes for large-scale wastewater treatment applications.

In summary, EC is a rapid but incomplete process, while EO is thorough but slow.^172^ Besides that, EC swiftly removes pollutants, albeit partially, whereas EO achieves full oxidation over an extended duration.^173^^,^^174^ Therefore, integrating EC with peroxicoagulation offers synergistic benefits, as the residual iron generated during EC can act as a catalyst for the peroxicoagulation process, enhancing the overall treatment efficiency.^174^

## Evaluation and optimization of EC systems

### Evaluation of EC performance

Due to the difference in experimental conditions, types of electrode materials, and contaminant sources, it is hard to compare results across studies without standardized performance metrics. Therefore, using the following mathematical equations to normalize data for cross-study comparisons is compulsory.

#### Removal efficiency

The removal efficiency, defined as the percentage of a particular effluent extracted from wastewater, is determined using Equation 22.^106^

(Equation 22) $$R\;\left(\%\right)=\frac{C_{0}-C_{t}}{C_{0}}\times 100\%$$

where R (%) denotes the effluent removal efficiency, while C_0_ and C_t_ represent initial and final effluent concentrations, respectively.

#### Synergistic effect

In the single-one-method process, performance depends on only the intrinsic variables of this method, while when two or more approaches are combined, the result often exceeds the sum of each individual result. Therefore, the synergistic effect (SE) between component methods should be scrutinized in performance evaluation and can be calculated via Equation 23. A positive SE value, where SE > 0, indicates a beneficial synergistic effect, highlighting the enhanced efficiency and effectiveness achieved through the integration of combined processes.^175^ Otherwise, if SE is negative, combining single-one methods into one method including multiple stages is meaningless0.

(Equation 23) $$\text{SE}=\left(\frac{\text{Removal}\;\text{efficiency}\;\text{of}\;\text{hybrid}\;A-B\;\text{method}\;\left(\%\right)}{\text{Removal}\;\text{efficiency}\;\text{of}\;\text{single}\;A\;\text{method}\;\left(\%\right)+\text{Removal}\;\text{efficiency}\;\text{of}\;\text{single}\;B\;\text{method}\;\left(\%\right)}-1\right)\times 100\%$$

#### Energy consumption

##### Internal energy

The work function (WF) of a metal is defined as the minimum energy required to transfer an electron from the metal anode to a point just outside its surface in a vacuum.^176^ This intrinsic property indicates the smaller WF value, the lower of energy consumption achieved during the electrochemical process. According to Table 4, silver is the most suitable candidate for an anode material in EC systems due to its rapid anodic dissolution properties.^177^ However, the economic feasibility of electrode materials is primarily determined by their cost, making material selection a critical factor for practical applications. Based on cost-effectiveness and WF value, Al and Fe are common materials in EC systems.

**Table 4:** The WF value of different metals in vacuum and water environments

Materials	WF in vacuum (eV)	WF in water (eV)
Al	4.25	3.00
Fe	4.31	3.02
Zn	4.24	2.77
Cu	4.70	3.46
Ag	4.00	2.77

Considering the values of the WF, the total energy required for the release of the electrode cation is known by Equation 24.^178^

(Equation 24) $$\text{EW}=n_{e}\times \text{WF}\times \frac{m_{\text{elec}}}{M_{\text{elec}}}\times N_{A}\left(\text{kJ}\right)$$

Where n_e_ is the number of free electrons of electrode metal, WF is the work function value (eV), m_elec_ (g) and M_elec_ (g/mol) are the mass and the atomic mass of the released electrode metal, respectively, N_A_ is the Avogadro constant (6.022 × 10^23^).

##### External energy

The total passed electric charge Q (C) that is affected by an applied voltage U (V) in the EC system, can be determined by integrating the current I (A), over time t (s), as described by Equation 25.

(Equation 25) Q=∫Itdt

The theoretical amount of ion production (or dissolved electrode metal) m_elec_ (g) is calculated via Faraday’s law (Equation 26).^61^

(Equation 26) $$m_{\text{elec}}=\frac{Q\times M_{\text{elec}}}{F\times n_{e}}$$

Where F denotes Faraday’s constant (96,485 C mol^−1^).

Besides that, the real-time dissolved amount of material from the anode is experimentally calculated by measuring the weight difference of the anode before and after the experiment.

The energy consumption W of the electrodes is calculated using Equation 27

(Equation 27) $$W=U\times I\times \frac{t}{V}\left(\text{kWh}\;m^{-3}\right)$$

Here U is the voltage (V), and V is the volume of electrolyte (m^3^).

#### Operation cost

Operation cost based on electricity and material costs is calculated by Equation 28.

(Equation 28) $$\text{Operation}\;\text{costs}=a\times W+b\times m_{\text{elec}}$$

Here, a and b represent the average price of electricity and electrode materials, respectively, and C_EC_ (kg m^−3^) denotes the quantity of electrode material required for treating 1 m^3^ of water.

#### Kinetic and diffusion investigations

The extensive use of simplified kinetic models in research can be attributed to the inherent complexity of mathematically describing the multitude of simultaneous physicochemical processes occurring in EC reactors. These processes include surface and bulk electrochemical and chemical redox reactions, dissociation reactions, hydrolysis, crystallization, coagulation, precipitation, co-precipitation, complexation, adsorption, flotation, and mass transfer.^179^ Regarding the kinetic modeling of EC, existing approaches predominantly employ empirical pseudo-first-order or pseudo-second-order equations to characterize pollutant removal as following Equations 29 and 30.^98^

Pseudo-first-order model

(Equation 29) $${\text{lnC}}_{t}=K_{1}t$$

Pseudo-second-order model

(Equation 30) $$\frac{1}{C_{t}}-\frac{1}{C_{0}}=K_{2}t$$

where C_0_ and C_t_ represent the initial and final effluent concentrations, respectively. K_1_ (min^−1^) and K_2_ (L mg^−1^ min^−1^) are the first- and second-order rate constants, respectively, while t denotes the treatment time (min). Plotting lnC_t_ and $\frac{1}{C_{t}}-\frac{1}{C_{0}}$ against time for each run yields a straight line, with the slope corresponding to K_1_ for the first-order rate and K_2_ for the second-order rate.

The diffusion rate coefficient, k_i_, is calculated based on the intraparticle diffusion model (Equation 31):^61^

(Equation 31) $$q_{t}=k_{i}t^{0.5}$$

where k_i_ is the rate constant, mg g^−1^ min^−0.5^.

### Optimization and modeling of EC systems by statistical and AI methods

Optimization and modeling of the EC process are important in scaling up to an industrial level and analyzing the techno-economic relationship of EC systems in water management. Due to the complex relationship between input variables and output results, optimization and modeling of EC systems under such conditions require several partial differential equations, which are often difficult to solve and involve many model parameters. The response surface methodology (RSM) and AI are well-known tools for the optimization and modeling of the response variables in EC systems. The following sections will discuss the advantages and disadvantages of each tool implemented in the evaluation of EC systems.

#### Statistical and mathematical methods

Because the output results in EC systems are affected by various input variables, the synergistic effect between these variables cannot be ignored. Therefore, statistical and mathematical methods are useful approaches to analyze and optimize the EC process. Design of experiment (DoE) encompasses a suite of advanced mathematical techniques for statistical modeling and systematic analysis, focusing on optimizing variables or factors to achieve desired outcomes.^178^^,^^179^ Among the diverse DoE methodologies, RSM is prominently used for studying the synergistic effect of independent input variables on output results via statistical and mathematical methods. In recent years, RSM has gained wide recognition as a tool for generating an experimental design matrix and improving various wastewater treatment processes, including filtration, adsorption, electrochemistry, and photodegradation.^180^^,^^181^^,^^182^^,^^183^ This method essentially uses a collection of mathematical and statistical approaches to design the experiments, develop the models, and finally maximize or minimize the predetermined responses to obtain the optimum operational conditions.^184^ Usually, in RSM, the regression model is implemented based on the experimental design and parameters are evaluated using response surface and contour plots.^185^

When the EC system includes many investigated input variables, a full factorial method is not reasonable due to excessive hands-on experiments, which increases operation costs. Therefore, this is crucial to minimize the number of experiment investigations and simultaneously maximize the accuracy of regression models showing the relationship between predicted output results and independent output variables. Two popular design methods, central composite design (CCD) and Box-Benken design (BBD) have been developed to reduce the number of hands-on experiments compared to full factorial design (FFD). In BBD, the experimental points are located on a hypersphere equidistant from the central point, while CCD is utilized to assess lower-order experimental effects including the following parts: the points of factorial design, the axial points, and the central point. BBD precisely fits the quadratic model, where removal combinations manifest at the center point and midpoints along the edges of the cube. This design avoids points at the corners of the cubic region formed by the lower and upper bounds of each variable. CCD is particularly advantageous for studying factor effects on responses and for optimization tasks, as it enables the creation of quadratic surfaces and enhances the estimation of effective parameters with fewer experimental runs. The number of experiments (N) required for the development of BBD and CCD is defined as follows Equations 32 and 33, respectively.^186^

(Equation 32) $$N=2k\times \left(k-1\right)+C_{0}$$

(Equation 33) $$N=2^{k}\times 2k+C_{0}$$

where k is the number of factors and C0 is the number of center points.

Using CCD to evaluate the EC performance is also widely used in various EC systems.^187^^,^^188^ For example, Arab highlighted the importance of electrode materials in microplastic removal by EC using RSM based on CCD.^189^ Nonetheless, in this study, to enhance the accuracy of CCD-RSM, more levels of input data such as current density, electrode spacing, and reactor design should be further studied. In another study, CCD was used to design the experiment runs of input variables such as electrolysis time, current density, pH, and initial concentration of effluents in evaluating dairy wastewater removal by coupled EC-AD approach.^190^ The obtained data were used to construct a regression model with a high R^2^ indicating a strong correlation between the experimental and predicted values.

However, due to complex processes including linear, nonlinear, and time-varying characteristics in EC systems, a quadratic linear regression model is inadequate for describing the systems’ nonlinearities.^191^ Hence, in the modeling, simulation, and optimization of processes, using phenomenological or traditional empirical models is not always the most appropriate solution. Under such circumstances, the modeling method based on AI models has higher reliability and wider coverage and has been widely used in process predictive control.

#### Artificial intelligence

Empirical (regression) modeling is an alternative approach to phenomenological modeling. Typically, a quadratic linear regression model is chosen, though it often fails to capture the nonlinearities inherent in the systems. Consequently, using phenomenological or conventional empirical models for modeling, simulating, and optimizing processes may not always be the best approach.^192^ Due to the complexity and diversity of these systems, modeling, simulation, and optimization present significant challenges. AI, a widely adopted technology, has made its way into chemical engineering research, offering effective solutions to complex and nonlinear problems.^191^^,^^193^^,^^194^ Thus, applying AI methods to predict the removal rate in EC reactors is expected to enhance both accuracy and speed. AI techniques, such as artificial neural networks (ANNs), adaptive neuro-fuzzy inference systems (ANFIS), support vector regression (SVR), and metaheuristic algorithms like genetic algorithms (GAs) and particle swarm optimization (PSO), have become attractive options for modeling and optimizing nonlinear processes.^195^^,^^196^^,^^197^^,^^198^ These data-driven models rely on empirical data and the relationships between process inputs and outputs rather than on process knowledge. To evaluate AI model performance, criteria such as mean square error (MSE), coefficient of determination (R^2^), and mean absolute percentage error (MAPE) are commonly used. MSE serves as the error function for training and validating hybrid models. These functions were calculated as:

$$\text{MSE}=\frac{1}{n}{\sum_{i=1}^{n}\left(y_{i}-\overset{\wedge }{y}\right)}^{2},R^{2}=1-\frac{\sum_{i=1}^{n}{\left(y_{i}-{\overset{\wedge }{y}}_{i}\right)}^{2}}{\sum_{i=1}^{n}{\left(y_{i}-{\bar{y}}_{i}\right)}^{2}},\text{MAPE}=\frac{100}{n}\sum_{i=1}^{n}\left|\frac{y_{i}-{\overset{\wedge }{y}}_{i}}{y_{i}}\right|$$

where $y_{i}$, ${\overset{\wedge }{y}}_{i}$, and $\bar{y}$ are the experiment-, predicted-, and the average values of the experimental data, respectively. The model possessing low MSE and MAPE and high R^2^ values shows good accuracy.

However, there are several challenges in using AI for EC systems, including issues with data quality, difficulty in understanding how models make decisions, high computational needs, and the risk of overfitting, which can affect accuracy and scalability. Also, integrating AI into existing systems requires expert knowledge, and the lack of standard guidelines makes it hard to get consistent results.

## Conclusion and perspectives

With the rapid increase in global population, addressing effectively the water crisis and sustainable water management are highly urgent. Endowed with cost-effective, simple operation and fewer secondary contaminants, EC has demonstrated potential in wastewater treatment. This paper systematically reviews the relationships between input parameters such as current, pH, electrode distance, electrolytes, and output results. However, some contaminants in wastewater using EC have not been completely eliminated as a weak point of the EC process. Therefore, to enhance the removal performance, coupling EC with supplementary methods such as adsorption, filtration, chemical oxidation, and electrochemical oxidation that can play as pre-treatment or post-treatment stage for EC, is highly recommended. To clarify the influences of the synergistic effect of several input variables and the removal efficiency, RSM provides a set of statistical and mathematical tools offering a systematic approach for modeling and analyzing complex EC processes. However, to comprehensively evaluate in a practical large-scale operation, the implementation of AI in modeling and automating EC systems is supportively encouraged.

The future perspectives of wastewater treatment using EC-based processes are suggested to discuss as follows.

(1) While numerous EC-based strategies widely focus on enhancing removal performance and reducing energy consumption, proper treatments of post-treatment EC sludge have not been effectively reported. The large amount of sludge generated after EC processes contains complex components with varying intrinsic properties. Therefore, suitable approaches for the sustainable treatment of sludge are highly desirable. Depending on different contaminant sources, the produced sludge of coupled EC approaches can be utilized effectively in various applications. For example, organic-rich sludge can undergo pyrolysis to produce biochar that further benefits agriculture and energy production. In addition, calcined sludge can be used as construction materials and adsorbents, helping to reduce the use of traditional raw materials. Nonetheless, ensuring the toxic and harmful levels of sludge is compulsory and remains a challenge to minimize any potential risks to human health and environment.
(2) With the rapid development of technologies for exploring sustainable energy sources, renewable electricity-driven EC processes for wastewater treatment have been proposed as an environmentally friendly alternative compared to conventional high-carbon footprint electricity supply. Energy conversion and storage devices have been used to power EC systems that address the geographic and seasonal mismatch of electricity supply and demand in remote areas where access to the electric grid is limited. Even though the price of those devices is affordable due to advancements in energy storage technologies, the scalability and long-term stability with high energy efficiency are insufficient to completely replace traditional electricity sources.
(3) Modeling and optimization are seen as a key component in scaling up EC process to the industrial level. AI has been championed as a tool for modeling the nonlinear EC system, which has complex relationships between input variables and output results. Therefore, AI implementation in the EC process not only helps to reduce operation costs but also benefits automative control. However, the predictive effectiveness and accuracy of AI models depend on the experimental database. Consequently, to achieve high accuracy in AI-EC systems, large and diverse datasets and a balance between AI-based predictions and real-time data need to be considered.

### Limitations of the study

This work has not presented details on electrode connections (monopolar parallel, monopolar series, and bipolar series), types of EC reactor designs, or types of EC systems (batch and flow systems).

## Acknowledgments

This work was supported by the project “Advancing Sustainable Wastewater Treatment through Electrocoagulation, AI, and Innovative Materials” (grant no. VUNI.2324.SG05) from VinUniversity, Vietnam.

## Author contributions

T.K.C.P., writing – original draft, writing – review & editing, visualization, conceptualization; P.L.N., conceptualization, writing – review & editing, and supervision; T.V.B.P., conceptualization, writing – review & editing, funding acquisition, and supervision.

## Declaration of interests

The authors declare no competing interests.

## Declaration of generative AI and AI-assisted technologies in the writing process

During the preparation of this work the authors used ChatGPT in order to improve readability and language of the work. After using this tool/service, the author reviewed and edited the content as needed and took full responsibility for the content of the publication.

## Supplemental information

Data S1. Elsevier’s Scopus database used to create Figure 2, showing the annual publications in the electrocoagulation field
